# Arginylation-Dependent Neural Crest Cell Migration Is Essential for Mouse Development

**DOI:** 10.1371/journal.pgen.1000878

**Published:** 2010-03-12

**Authors:** Satoshi Kurosaka, N. Adrian Leu, Fangliang Zhang, Ralph Bunte, Sougata Saha, Junling Wang, Caiying Guo, Wei He, Anna Kashina

**Affiliations:** 1 Department of Animal Biology, School of Veterinary Medicine, University of Pennsylvania, Philadelphia, Pennsylvania, United States of America; 2 University Laboratory Animal Resources, University of Pennsylvania, Philadelphia, Pennsylvania, United States of America; 3 Janelia Farm, Ashburn, Virginia, United States of America; 4 Gene Targeting and Transgenic Facility, University of Connecticut Health Center, Farmington, Connecticut, United States of America; California Institute of Technology, United States of America

## Abstract

Coordinated cell migration during development is crucial for morphogenesis and largely relies on cells of the neural crest lineage that migrate over long distances to give rise to organs and tissues throughout the body. Recent studies of protein arginylation implicated this poorly understood posttranslational modification in the functioning of actin cytoskeleton and in cell migration in culture. Knockout of arginyltransferase (*Ate1*) in mice leads to embryonic lethality and severe heart defects that are reminiscent of cell migration–dependent phenotypes seen in other mouse models. To test the hypothesis that arginylation regulates cell migration during morphogenesis, we produced *Wnt1*-Cre *Ate1* conditional knockout mice (Wnt1-Ate1), with *Ate1* deletion in the neural crest cells driven by *Wnt1* promoter. Wnt1-Ate1 mice die at birth and in the first 2–3 weeks after birth with severe breathing problems and with growth and behavioral retardation. Wnt1-Ate1 pups have prominent defects, including short palate and altered opening to the nasopharynx, and cranial defects that likely contribute to the abnormal breathing and early death. Analysis of neural crest cell movement patterns in situ and cell motility in culture shows an overall delay in the migration of *Ate1* knockout cells that is likely regulated by intracellular mechanisms rather than extracellular signaling events. Taken together, our data suggest that arginylation plays a general role in the migration of the neural crest cells in development by regulating the molecular machinery that underlies cell migration through tissues and organs during morphogenesis.

## Author Summary

Formation of many organs during development depends on the coordinated migration of individual cells and cell layers throughout the embryo. The majority of migrating cells originate from the neural crest lineage that gives rise to peripheral neurons, ganglia, pigment cells, and craniofacial structures, as well as parts of other organs in the body. Recent studies have implicated arginylation—a poorly understood protein modification—in the regulation of basic mechanisms that underlie cell migration. Here we test the role of arginylation in neural crest cell migration during mouse development by constructing and examining a mouse model with arginylation-deficient neural crest cells. We find that these mice die at or soon after birth and exhibit severe defects in the development of distinct neural crest-derived structures. Our findings uncover a previously unknown mechanism of the regulation of neural crest cell migration during development, and shed light on general principles of neural crest migration in vivo.

## Introduction

Coordinated cell migration during development is crucial for tissue and organ morphogenesis from early gastrulation to adulthood. The largest cell populations that are capable of long-range migration at different developmental stages originate from the neural crest lineage. Neural crest cells are of mesenchymal morphology and migrate from the trunk into different areas of the developing embryo. These cells express a distinct subset of markers, including *Wnt1* and others [1]–[8], at or before the onset of migration.

Recent studies of protein arginylation demonstrated an essential role of this poorly understood posttranslational modification in mammalian embryogenesis and suggested that arginylation is a previously unknown major signaling mechanism that regulates multiple physiological pathways. Knockout of arginyltransferase (*Ate1*) in mice leads to embryonic lethality and severe cardiovascular defects, including abnormal heart septation, underdeveloped myocardium, and impaired angiogenesis [9]. Remarkably, all these phenotypes resemble the phenotypes seen in the mouse models with knockouts of various genes implicated in cell migration, leading to the hypothesis that the mechanisms underlying cell migration may be the primary targets for regulation by arginylation (see [10] for review). It has been found that a large number of proteins in vivo are arginylated [11]–[23], including a prominent subset of cytoskeletal targets that play direct mechanistic roles in cell migration. Arginylation of beta actin in cultured fibroblasts regulates lamella formation and the structure of the cell leading edge [24]. Other proteins involved in cell adhesion and migration, such as talin, spectrin, filamin, myosin, etc. are also arginylated in different mouse tissues [23]. All these data suggest that arginylation may be a general mechanism of the regulation of cell movement in different physiological events, however the role of arginylation in directional cell migration in culture and during embryonic development has never been studied before.

To test the hypothesis that arginylation regulates cell migration during morphogenesis, we produced and analyzed a conditional knockout mouse model with *Ate1* deletion driven by neural crest-marking *Wnt1* promoter (Wnt1-Ate1 mouse line). These mice exhibit perinatal lethality and severe morphogenesis defects resulting from poorly developed neural crest-derived structures, suggesting that *Ate1* indeed regulates the migration of neural crest cells that give rise to these structures in embryogenesis. Studies of cell migration patterns in embryos and in culture show that *Ate1* knockout results in an overall delay in the migration, likely regulated at the intracellular level, and that *Ate1* knockout cells co-cultured with wild-type tend to ‘ride’ on the migrating cells rather than move on their own. Taken together, our data indicate that arginylation regulates tissue and organ morphogenesis by affecting the intracellular mechanisms that drive the migration of the mesenchymal cells of the neural crest lineage.

## Results

### Generation of Wnt1-Ate1 mice

To produce an *Ate1* conditional knockout we first generated an ‘*Ate1*-floxed’ mouse line with the first three exons of the *Ate1* gene flanked by LoxP sites (Figure S1). We have previously shown that exons 1 and 2 are essential for the formation of all four *Ate1* isoforms [25] and that deletion of the region encoded by these exons from the *Ate1* sequence leads to the abolishment of *Ate1* activity in yeast complementation assays [26]. Control experiments (data not shown) confirmed that deletion of the exons 1–3 using the Cre-driven recombination in the *Ate1*-floxed line resulted in embryonic lethality similarly to the previously described *Ate1* knockout [9],[27]. To produce a neural crest-specific *Ate1* knockout, we crossed the *Ate1*-floxed line with a commercial mouse strain (*Wnt1*-Cre) where Cre recombinase is expressed under neural crest-inducing *Wnt1* promoter, resulting in *Ate1* deletion in subsequently derived neural crest and some other cell types. These mice, termed Wnt1-Ate1 mice, were used in the present study.

To confirm the efficiency of the *Ate1* knockout in these mice, we analyzed the *Ate1* protein expression by immunohistochemistry of the sagittal sections of E12.5 and E16.5 Wnt1-Ate1 embryos probed with rat monoclonal antibody that recognizes all four *Ate1* isoforms (see Figure S2 for antibody characterization and Figure S3 and Figure S4 for embryo staining). In control embryos Ate1 expression was observed in most tissues and organs (data not shown), consistent with our previous data that *Ate1* is expressed throughout the embryo [9],[25]. In contrast, Ate1 expression in the conditional knockout mice was prominently excluded from the midbrain region and the enteric nerves, as well as parts of the peripheral nervous system (Figure S3 and Figure S4), the structures that are derived from *Wnt1* expressing cells [6],[28]. No prominent areas with missing Ate1 expression were observed anywhere else in the embryo, however it is possible that *Wnt1*-expressing *Ate1* knockout cells in other areas (such as, the somite regions along the back) mixed with other cell populations, making them difficult to detect. It is also possible that in such areas the normal levels of *Ate1* are reduced, making the knockout cells poorly distinguishable from the background. To address this possibility and further confirm the efficiency of the Cre transgene expression, we crossed *Wnt1*-Cre mice to the R26R Rosa reporter mouse strain, in which prominent LacZ expression occurs in all Cre-expressing tissues [29]–[32]. X-gal staining of *Wnt1*-Cre embryos at E9.5 showed that Cre expression occurred as expected, with the majority of staining observed in the head and the somite region (Figure S14).

### Wnt1-Ate1 mice exhibit perinatal lethality

Unlike the complete *Ate1* knockout mice that die just past mid-gestation (E12.5–E14.5) [9],[27], Wnt1-Ate1 mice survived until birth and exhibited perinatal lethality (Table 1). Over 60% of the pups died on the day of birth (P0), and another 24% died during the following 3 weeks, with less than 13% of mice surviving to adulthood. Such a variability in the mortality rate could be explained by the variability in the migratory patterns of neural crest cells between individual embryos (see below).

**Table 1:** Death/survival rates of Wnt1-Ate1 mice.

Numbers (%) of Wnt1-Ate1 mice			
born	died at		survived
	P0	P1–P21	
**125 (100.0)**	**79 (63.2)**	**30 (24.0)**	**16 (12.8)**

**Figure 1:**
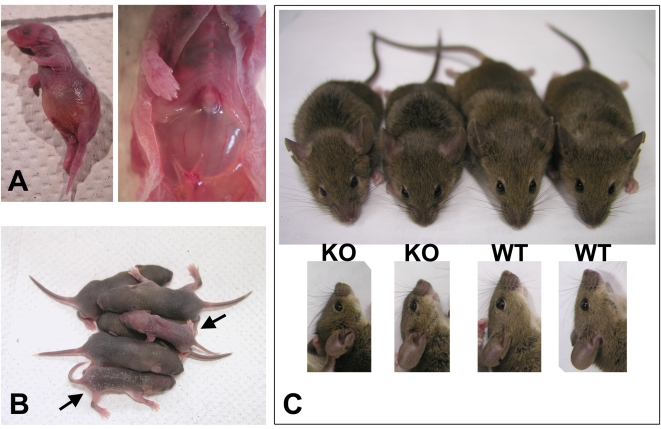
Wnt1-Ate1 mice have perinatal lethality. (A) A Wnt1-Ate1 pup that died at birth from breathing defects as shown in the Video S1. In such pups labored breathing leads to bloating (left) and prominent air accumulation in the stomach (right). (B) Wnt1-Ate1 pups that survive after birth (indicated by arrows) shown at postnatal day 5 (P5) are smaller than their wild-type littermates and are significantly less active at day 9 (Video S2), suggesting growth and behavioral retardation. (C) Wnt1-Ate1 (KO) mice that survive to adulthood are smaller than control (WT, top) and have short snouts and abnormally shaped skulls (bottom).

The conditional knockout mice that died at P0 had severe breathing problems. Unlike their littermates they breathed frequently and irregularly and appeared to be gasping as if from the lack of air (Video S1). Within a few hours, these mice became bloated, with abnormally enlarged stomachs, and died (Figure 1A, left). Post-mortem dissection showed large amounts of air in their stomach (Figure 1A, right), suggesting that the air was misdirected there from the respiratory tract, possibly through the digestive system, and accumulated due to extensive breathing.

Wnt1-Ate1 mice that died at later stages after birth (mostly within the first week, Table 1) showed no visibly abnormal breathing, however these mice appeared severely retarded, with delayed growth and significantly smaller body size compared to their littermates (Figure 1B) and apparent inability to actively move and explore the surrounding space (Video S2). Their appearance and behavior could be explained by malnutrition caused by non-lethal alterations in their ability to breathe and feed compared to their wild-type littermates. It is also possible, however, that these defects were caused by additional physiological and/or neurological abnormalities, caused by *Ate1* deletion in other neural crest cell populations.

The remaining 12.8% of Wnt1-Ate1 mice were able to grow to adulthood. These mice were fertile and able to produce healthy, surviving pups, however they were consistently smaller than their littermates and had visible facial abnormalities (short snouts and abnormally shaped skulls, Figure 1C). Thus, despite the variations in the severity of the Wnt1-Ate1 phenotype, over 87% of the conditional knockout mice died at or soon after birth, and all of them exhibited varying degrees of defects.

### Wnt1-Ate1 mice have craniofacial defects

It has been previously found that mouse knockouts of the genes implicated in neural crest cell migration and neural crest-dependent morphogenesis are often accompanied by the defects in palate and other craniofacial structures that originate from the neural crest cell lineage [33]–[36]. Palate defects in particular are known to correlate with breathing problems. It is hypothesized that shortened or cleft palates affect the separation between the digestive and respiratory tract, causing the inhaled air to be directed to the stomach rather than the lungs and resulting in air accumulation in the stomach and lethality similar to that seen in Wnt1-Ate1 mice at P0 [35],[37],[38]. To test whether Wnt1-Ate1 mice have defects in the palate and/or surrounding structures, we performed postmortem analysis of the pups that died at P0 with breathing abnormalities, by removing the lower jaw and visually analyzing the throat and the roof of the oral cavity.

**Figure 2:**
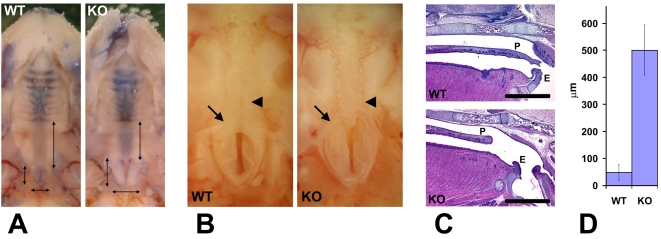
Wnt1-Ate1 mice have palate defects. (A) Observation of the roof of the mouth of control (WT) and Wnt1-Ate1 (KO) newborn pups at P0 shows a shorter soft palate area (longer vertical arrow on the right in each image) and an enlarged nasopharynx entrance (two shorter perpendicular arrows on the left and bottom in each image). Heads have been contrasted with trypan blue dye for better observation. 5 wild-type and 5 Wnt1-Ate1 pups were analyzed. (B) Closer view of the nasopharynx entrance (arrow) and the surrounding area in a control (WT) and aWnt1-Ate1 (KO) newborn pup at P0 with the missing soft palate (arrowhead). (C) Sagittal sections through the palate areas of the control (WT) and Wnt1-Ate1 (KO) newborn pups at P0. While in control the soft palate (P) reaches all the way to the epiglottis (E), in the mutant the palate is shorter and leaves a large gap of exposed tissue at the back of the throat. Scale bar, 1 mm. 4 wild-type and 5 Wnt1-Ate1 pups were analyzed. (D) Quantification of the distance from the end of the palate to the epiglottis in all the examined pups at P0 (4 wild-type and 5 mutants). In wild-type, the average distance was 48.6+/−28.9 (SEM), and in the mutants this number increased over 10-fold to 501.0+/−93.7 (SEM), indicating a significant overall shortening of the tissue of the soft palate.

Several defects were observed during this analysis. First, the majority of the analyzed mutant pups had an abnormally large entrance to the nasopharynx and a reduced area normally covered by the soft palate (denoted by two shorter perpendicular arrows and one longer arrow in Figure 2A, respectively). In control animals (Figure 2A, left) the entrance to the nasopharynx was tightly shut and resembled a vertical slit surrounded by a small area of soft tissue. In the mutant animals (Figure 2A, right), the nasopharynx entrance appeared partially opened, assuming a triangular shape with a larger surrounding area, suggesting that this structure was prevented from closing either because of being structurally defective, or because the musculature that controls it did not operate properly. Closer observations (Figure 2B) showed that while in control mice (left) the entrance to the nasopharynx was partially closed by the soft palate, in the mutants (right) the soft palate in this area was either short (not shown) or missing (Figure 2B), leaving a gap that would be expected to permit the air to travel unrestrictedly throughout the passages connected to the oral cavity in this area.

To confirm that the defects we see are indeed palate defects, we fixed the newborn Wnt1-Ate1 pups and their control littermates and sectioned them sagittally through the middle, in the area where the palate appears as the horizontal line separating the oral and the nasal cavity. Consistent with our morphological observations, palates in all the conditional knockout pups analyzed at P0 appeared short, unable to reach the area of the throat where the separation between the trachea and the esophagus occurs (Figure 2C). The average distance between the end of the palate and the epiglottis in the mutant mice was approximately 10 times larger than that in control (Figure 2D). Thus, Wnt1-Ate1 knockout mice, similar to other mouse models with neural crest migration defects, have severe malformations of the soft palate that likely cause the breathing problems and misdirected air flow resulting in early postnatal lethality (Figure 1A and Video S1).

Since Wnt family genes, in addition to the neural crest, have been implicated in the functioning of other organs, including lungs [39],[40], we tested whether, in addition to the palate, other defects in Wnt1-Ate1 mice may contribute to the breathing problems and/or early lethality. Histological examination of the sagittal sections of the Wnt1-Ate1 embryos at P0 revealed no prominent abnormalities in the major organs or structures throughout the body (data not shown). Lungs in the Wnt1-Ate1 mice appeared collapsed compared to the wild-type, with little or no air in the alveolae and hemorrhaging in the air passages, however no visible lung defects were observed in the E16.5, E18.5 and E19.5 embryos recovered before birth that did not have a chance to breathe (data not shown). Therefore, given that Wnt1-Ate1 pups have breathing abnormalities that may indirectly affect the lungs, it appears unlikely that these mice have an independent structural lung defect.

**Figure 3:**
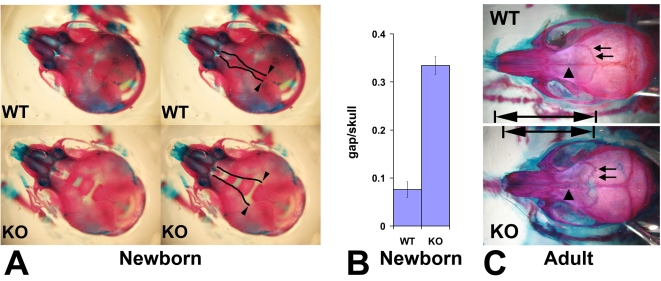
Wnt1-Ate1 mice have defects in the frontal bones. (A) Top view of the skulls of the control (WT, top) and Wnt1-Ate1 (KO, bottom) pups at P0 stained with alizarin red S and alcian blue 8GS show a significant gap on top of the skull in the mutant pups, due to the significantly reduced frontal bones. Left images show the same pictures as those shown on right with the edges of the gap outlined. Arrowheads indicate the point of the opening of the gap used for the measurements shown in (B). (B) Measurement of the ratio of the gap width to skull width along the same axis (gap/skull) at P0 show that in the mutant (KO) unlike the control (WT) the gap occupies on average more than 1/3 of the skull (ratio 0.334+/−0.018 (SEM, n = 5) in the mutant vs. 0.076+/−0.016 (SEM, n = 12) in wild-type). (C) Skulls of adult Wnt1-Ate1 mice compared to their littermate controls show frontal bone abnormalities, including abnormal shape, incomplete cranial suture (arrows), and abnormal suture between the frontal bones (arrowheads). 2 wild-type and 2 mutant animals were analyzed.

Since neural crest cells contribute to the formation of bone and cartilage in the head, we next examined the skeletons of newborn Wnt1-Ate1 and control mice at P0 by staining with alizarin red S and alcian blue 8GS that interact with bones and cartilage to color them red and blue, respectively [41] (Figure 3). While the overall bone structure and skeletal architecture appeared normal in Wnt1-Ate1 mice (data not shown), prominent abnormalities were seen in the development of the frontal bones, the neural-crest-derived parts of craniofacial skeleton that contribute to the top of the skull [32],[42]. In control mice, frontal bones came close together, leaving only a narrow slit along the top of the skull (Figure 3A, top images). In contrast, in the mutant mice, frontal bones appeared smaller and narrower and were unable to meet on top of the skull, leaving a wide gap that exposed the cranial cavity beneath (Figure 3A, bottom images). Measurements of the ratios between the width of the gap and the width of the skull showed that in the mutant the gap occupied on average almost 1/3 of the skull width, an area almost 4 times larger than in wild-type (Figure 3B). This defect was observed in all the analyzed mutants, even those that did not exhibit breathing defects at birth.

To test whether frontal bone defects are also seen in the Wnt1-Ate1 mice that survive to adulthood, we euthanized several adult Wnt1-Ate1 animals and matching wild-type controls and stained their skeletons similarly to the way described above for the newborn mice. Consistent with the defects seen in the newborns, adult surviving Wnt1-Ate1 mice had short, deformed frontal bones that had altered shape and rougher outline and often appeared incompletely closed (arrows in Figure 3C) and unable to meet in the middle (arrowheads in Figure 3C). Nasal bones also appeared shortened, leading to the overall shortening of the snouts as seen in the intact animals (Figure 1C). Therefore, some neural crest-derived parts of the craniofacial skeletons are affected in Wnt1-Ate1 mice regardless of the severity of other phenotypic changes seen in these mice.

Since, in addition to craniofacial structures, Wnt1-Ate1 mice also show prominent *Ate1* deletion in the enteric neurons and some deletion in the peripheral nervous system (Figure S3 and Figure S4), we tested whether the mutants have abnormal distribution of peripheral nervous system structures and gut neurons that might indicate defects in gut innervation often associated with neural crest-related developmental abnormalities (reviewed in [43]–[45]) by staining embryo sections and whole mount guts excised from E16.5 Wnt1-Ate1 embryos with antibody to the neuron projection marker beta-III tubulin. This staining revealed no abnormalities in the peripheral nervous system (Figure S4) or gut neuronal network (Figure S5), suggesting that the peripheral nervous system and enteric neurons, despite being *Ate1*-deficient, were able to position normally during embryogenesis.

### Wnt1-Ate1 neural crest cells show defects in migration patterns during development

**Figure 4:**
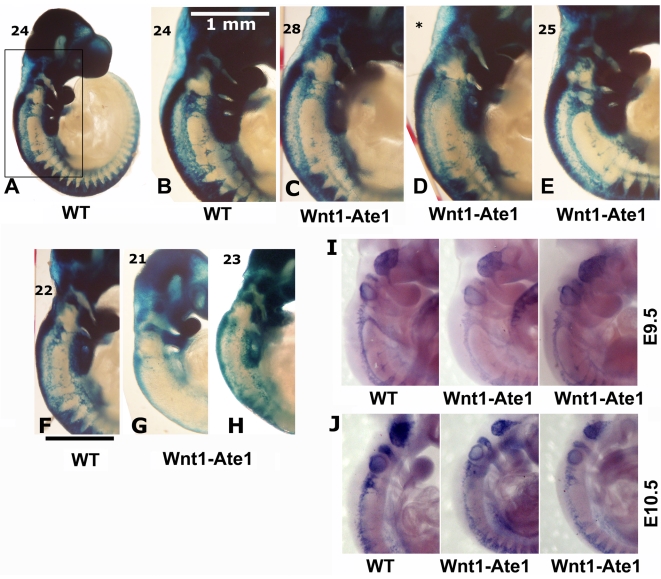
Wnt1-Ate1 mice have defects in neural crest cell migration. (A–H) X-gal staining of E9.5 control (A,B) and Wnt1-Ate1 (C–E) embryos derived from Wnt1-Ate1-R26R mouse line grouped by somite count (indicated on the top left for each embryo). A, lower magnification image of a control embryo at 24 somite stage. (B–E), back areas of different wild-type and Wnt1-Ate1 embryos with higher somite count, corresponding to the region boxed in (A), showing different neural crest migration patterns as described in the text. For Wnt1-Ate1, three littermates are shown, illustrating different pattern and severity of defects as described in the text; asterisk indicates the embryo, for which somite count was not performed and the staging relied on the comparison with its littermates shown on both sides. (F–H), back areas of wild-type and Wnt1-Ate1 embryos with lower somite count. Bar, 1 mm, for the images shown in (B–H). 10 wild-type and 10 mutant embryos were analyzed. See Figure S14 for whole embryo views. (I,J) In situ hybridization of E9.5 (I) and E10.5 (J) control and Wnt1-Ate1 embryos using *Sox10* neural crest marker. 2 wild-type and 2 Wnt1-Ate1 embryos were analyzed for each developmental stage. See Figure S15 for whole embryo views.

To address the question whether Wnt1-Ate1 mice exhibit any defects in neural crest cell migration, we used the Wnt1-Ate1-R26R reporter conditional knockout line and analyzed the distribution of LacZ -expressing cells in wild-type and Wnt1-Ate1-R26R embryos at E9.5. Several litters, whose embryonic stage was determined by counting the somites, were analyzed, and the comparisons were made between embryos with comparable somite numbers. In the analyzed embryos, the somite numbers ranged between 22 and 26 in wild-type and between 21 to 28 in Wnt1-Ate1, consistent with the expected somite numbers at this stage. While the X-gal staining of the migrating neural crest cells in these embryos, especially with the larger somite numbers, was heavily masked by staining of other organs originating from *Wnt1*-expressing cells, such as midbrain (Figure S14), cell migration patterns could be clearly observed in the somite regions and near the pharyngeal arches, where the migrating cells appeared as streams of LacZ-expressing ‘dots’ arranged in different patterns from the back to the front of the embryo (Figure 4). In wild-type (Figure 4A and 4B), prominent populations of cells migrated from back to front as continuous lines (from the third pharyngeal arch down to the somites) and as triangular ‘streams’ directed toward the third and fourth pharyngeal arches and along each of the somites. In Wnt1-Ate1, several of these migration patterns were affected with different degrees of severity. Embryos with 24+ somites had altered cell distribution in the streams migrating toward the third and fourth pharyngeal arches and in the line migrating over the upper area of the trunk, which appeared diffuse, with fewer cells present in those areas (Figure 4C and 4D). In some embryos (Figure 4E), migration toward the pharyngeal arches appeared normal, but X-gal staining in the upper trunk area appeared weaker than control, indicating a reduced number of migrating cells in that area. In the embryos at a slightly earlier developmental stage (21–23 somites, Figure 4F–4H) these differences were more obvious, resulting in much lower overall levels of the X-gal-stained cells visible in these areas. Such extremely affected embryos also appeared smaller than wild-type embryos or knockout embryos with less severe phenotypes (see Figure S14).

To further confirm this result, we performed in situ hybridization of whole mount embryos for the neural crest marker *Sox10*. Analysis of embryos at E9.5 and E10.5 showed similar changes in the migration patterns in Wnt1-Ate1 mice compared to control (Figure 4I and 4J and Figure S15). Finally, to obtain an independent confirmation of altered migration in *Ate1* knockout neural crest cells, we isolated neural crest explants from E8.5 Wnt1-Ate1-R26R mice, and observed the migratory patterns of LacZ-expressing cells after incubation for 48 hourrs in culture (Figure S6). While in control explants (Figure S6, left panels), many of the LacZ-expressing cells during this time emigrated from the original cell mass and traveled to the periphery of the expanding explant as groups or individual cells, in the knockout LacZ-expressing cells traveled to a shorter distance as streams of cells mostly connected to the original explant, without venturing out on their own (Figure S6, right panels). All of the examined explants behaved consistently with each other, suggesting that this altered migration pattern occurs universally in response to *Ate1* knockout. Therefore, consistent with the situation in situ, *Ate1* knockout in the neural crest cells results in impairment of their migration in culture.

Since most of the developmental defects observed in Wnt1-Ate1 knockout mice are related to the size reduction of the structures that are normally derived from the neural crest cells (such as palate or frontal bones), it is possible that in addition to decreased cell migration some of these defects are due to other reasons, such as decreased proliferation or increased apoptosis in the *Ate1* knockout neural crest cells. To test for possible increase in apoptosis, we used TUNEL assay to stain E9.5 Wnt1-Ate1 embryos (Figure S7) or E12.5 *Ate1* knockout embryos (not shown) and found no differences in the amount or distribution of the stain in the wild-type and knockout littermates. We also stained sections of Wnt1-Ate1 embryos with antibodies to the apoptotic cell marker cleaved caspase 3 and found no differences in the staining intensity or patterns between wild-type and Wnt1-Ate1 embryos (see Figure S8 for representative images).

To test for possible changes in cell proliferation, we stained embryo sections for the cell proliferation marker phospho-histone H3 and found no differences in the staining intensity or distribution between wild-type and Wnt1-Ate1 embryos (Figure S9). To further test this possibility, we isolated neural crest explants from E8.5 and E9.5 *Ate1* knockout littermate embryos and, after two days in culture, labeled the actively proliferating cells with 5-bromo-2-deoxyuridine (BrdU). No difference was found between the numbers or distribution of actively proliferating cells in wild-type and *Ate1* knockout explants (Figure S10).

These results suggest that *Ate1* knockout does not cause changes in the rates of cell proliferation or apoptosis during embryogenesis, pointing to the fact that the defects observed in Wnt1-Ate1 knockout mice are due primarily to the impairment in neural crest cell migration.

### *Ate1* knockout cells of mesenchymal morphology migrate abnormally in culture

To further characterize impairments in cell migration induced by *Ate1* knockout, we analyzed the motility of cultured *Ate1* knockout fibroblasts, derived from the back portion of the E12.5 *Ate1* knockout embryos and immortalized by continuous passaging in culture as described in [9],[24]. This model was chosen as one of the closest cell culture models of neural crest cell migration. Indeed, these fibroblasts are mesenchymal cells derived from the back portion of the embryos where the majority of the migrating neural crest cells are also found, and they are morphologically indistinguishable from the cells composing the neural crest explants in culture. Therefore, such fibroblasts can be expected to behave in the same manner as the neural crest cells in terms of their motile properties, and using these cells as a model in culture makes it possible to perform much more detailed tests than possible with neural crest explants.

*Ate1* knockout fibroblasts have been previously shown to have a defective lamella [24], however no changes in the speed or directionality of their migration have been reported. To test whether these cells move slower than wild-type, we performed scratch wound assays by removing an area of a dense cell monolayer and taking time lapse images of cells moving from the dense area to the scarce. The observations were performed over long periods of time similar to the estimated duration of many neural crest-dependent migratory events in development (10–15 hours).

**Figure 5:**
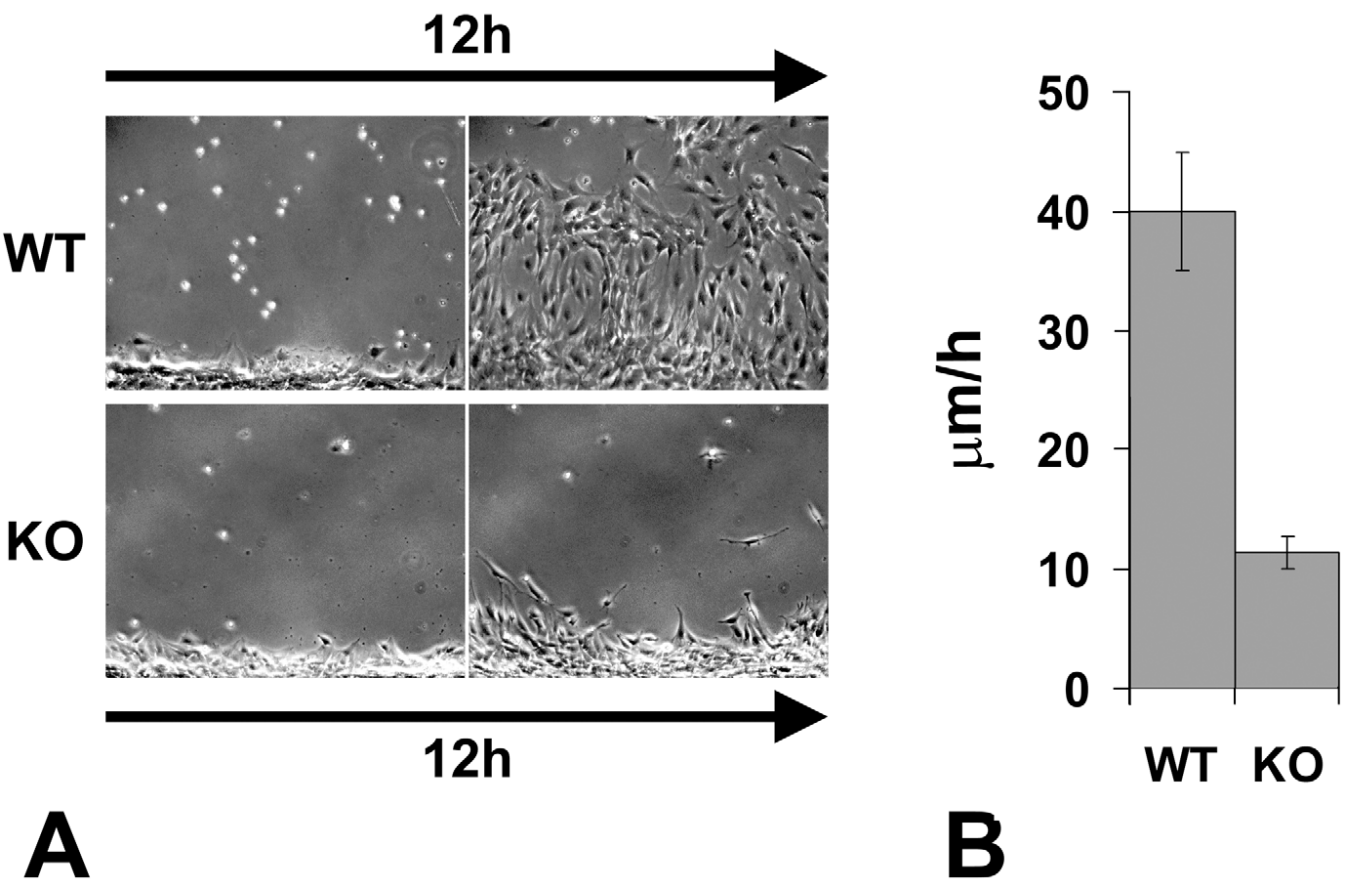
Knockout of the arginyltransferase *Ate1* in the mesenchymal cells results in a significant reduction in their migration speeds. (A) The first and the last frame of a time lapse series of a wild-type (WT) and *Ate1* knockout (KO) fibroblast monolayer moving into the wound. See Video S3 and Video S4 for the corresponding time lapse series. Bar, 200 µm. (B) Quantification of the average migration speed in wild-type (WT) and *Ate1* knockout (KO) cultures calculated from the time lapse series similar to the one shown in (A). In wild-type average migration speed was 39.97+/−4.90(SEM), n = 2; in the knockout the speed was almost 4 times slower at 11.34+/−1.32(SEM), n = 4.

While individual *Ate1* knockout cells during shorter stretches of time were capable of moving at speeds similar to wild-type (not shown), the migration speed of the *Ate1* knockout cell monolayers over continuous periods of time was nearly 4 times slower than that of wild-type cells (average speed of 11.5 µm/h compared to 41.3 µm/h in wild-type) (Figure 5, Video S3 and Video S4). This difference constitutes a significant delay in the overall migration, and, when transferred to an in vivo environment of a developing embryo would be likely to create significant morphogenic defects.

**Figure 6:**
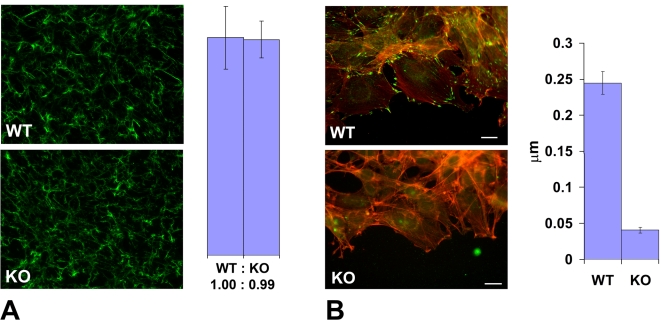
*Ate1* knockout affects cell attachment to the substrate via intracellular and not extracellular mechanisms. (A) Left, extracellular fibronectin staining in a dense monolayer of wild-type (WT) and *Ate1* knockout (KO) cultured fibroblasts shows no difference between the two cultures. Right, quantification of the fibronectin level in the two cultures measured as average gray value in the entire field of view confirms that there is no difference between WT and KO cells in the amount of extracellular fibronectin. Bars show the ratio between WT and KO and error bars represent the average of the measurements in 10 different fields of view in each culture. (B) Left, an overlay of the fluorescence staining of the edge of the cell monolayer moving into the wound co-stained with rhodamine-phalloidin (red) to visualize the actin filaments and anti-paxillin (green) to visualize the focal adhesions. Right, quantification of the number of focal adhesions per µm of the wound edge shows that the number of prominent focal adhesion in wild-type exceeds that in the knockout by over 5-fold. Error bars represent the measurements in 21 and 18 different images in WT and KO, respectively. Bar, 20 µm.

Cell migration in culture and in situ is mediated by attachment to the substrate and forming a connection between the intracellular actin cytoskeleton and the extracellular matrix via focal adhesions. To test whether the slow migration speeds in *Ate1* knockout cells were due to their impaired adhesion on the intra- or extracellular side, we first tested whether these cells are capable of creating a local extracellular environment that favors migration. To do this, we stained wild-type and *Ate1* knockout non-permeabilized cell monolayers for fibronectin, a major extracellular matrix component that directs the migration and adhesion of the mesenchymal cells and is secreted by these cells in culture and in situ (reviewed in [46]). No differences were found in the amount or distribution of fibronectin per area in each culture (Figure 6A), suggesting that *Ate1* knockout cells are capable of creating and utilizing the same local extracellular environment as wild-type. Thus, the migration defects in *Ate1* knockout cells do not originate at the local extracellular level.

We next tested whether the intracellular defects in *Ate1* knockout cells could be responsible for slower migration speeds. To do this, we compared the number of focal adhesions (the structures that connect cells to the extracellular matrix and thus are responsible for cell movement) at the edge of the cell monolayer migrating into the wound in wild-type and *Ate1* knockout cells by staining these cells with three different focal adhesion markers, paxillin, talin, and focal adhesion kinase (FAK) (Figure 6B and Figure S11). While in wild-type cultures cells moving into the wound developed prominent focal adhesions that appeared firmly anchored to the substrate (Figure 6B, top panel, and Figure S11, left panels WT), in *Ate1* knockout cells focal adhesions appeared smaller and scarcer, sometimes difficult to detect (Figure 6B, bottom panel, and Figure S11, right panels). This decrease in focal adhesion size and number was accompanied by a reduced level of paxillin but did not correlate with the changes in talin and FAK protein level (Figure S11, middle panels KO), suggesting that the decrease in focal adhesion constituted a genuine structural defect rather than a secondary effect of down-regulation of any of these markers. Manual counting of the number of prominent elongated paxillin-containing structures per µm of the wound edge, or the area of talin-containing structures per leading edge in each cell, revealed an almost 10-fold reduction in the focal adhesion area and number in *Ate1* knockout cells compared to wild-type (Figure 6B and Figure S11, right panels). Such a prominent difference is highly likely to severely affect both the cell's ability to attach to the substrate and the resulting cell migration. Thus, *Ate1* knockout-dependent impairment in cell migration likely originates at the intracellular level.

To further study the focal adhesion defects in *Ate1* knockout cells we quantified the total area of talin and paxillin-containing focal adhesions in single wild-type and *Ate1* knockout cells, and found that, consistent with the results obtained in the moving cell monolayer, the focal adhesion area in single cells is significantly reduced (Figure S12). This result suggests that the focal adhesion defects in *Ate1* knockout cells are the intrinsic property of these cells and not the secondary result of the impaired movement of these cells along the substrate. Finally, to corroborate this observation with the situation in situ, we stained embryo sections for talin and quantified the staining intensity in the migrating subpopulations of neural crest cells at the sides of the neural tube (Figure S13). While this assay measures not only talin localized to the adhesion sites but the total talin level in the tissue (which is similar in wild-type and knockout, as seen in Figure S11), the un-localized talin is expected to show a reduced, more diffuse signal compared to talin bound at the focal adhesion sites. Consistent with this hypothesis and the results of focal adhesion quantification in culture, the talin staining in situ was reduced in Wnt1-Ate1 embryos by a small but statistically significant number (Figure S13), suggesting that migrating neural crest cells in these embryos have focal adhesion defects that may prevent them from proper attachment and movement through the tissues during embryogenesis.

Unlike in the complete knockout, when cells migrate in situ in a conditional mouse model, in many cases knockout cells are found in the immediate vicinity of the wild-type and are expected to co-migrate during different developmental events. Therefore, the straight comparison between wild-type and *Ate1* knockout cell movements does not necessarily reflect the complex situation in which these cells co-exist in situ, potentially affecting each others' migration. To test the motility of *Ate1* knockout cells in the environment of the surrounding wild-type cells, we performed wound healing migration assays of wild-type cells co-cultured with *Ate1* knockout cells labeled with stably transfected GFP. Control experiments showed that GFP-expressing cells moved at rates similar to the regular *Ate1* knockout cells when cultured separately from wild-type (data not shown).

**Figure 7:**
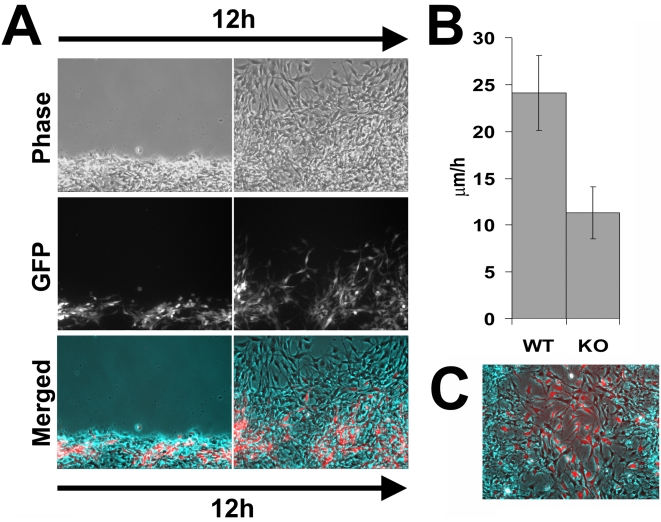
*Ate1* knockout cells co-cultured with wild-type move with speeds closer to the wild-type cells than when cultured individually. (A) Phase contrast (top), fluorescence (middle), and overlayed (bottom) images of the first and last frame of a 12-hour time lapse series of GFP-labeled *Ate1* knockout cells co-cultured with wild-type moving into the wound. Wild-type cells quickly bypass the *Ate1* knockout cells initially found at the wound edge and ‘lead’ for the rest of the time lapse, consistent with their faster migration speeds. *Ate1* knockout cells lag behind, often ‘riding’ on the wild-type cells rather than moving on their own, however they are able to cover greater distance over the 12 hours than in individual cultures (Figure 5). See Video S5 for the merged time lapse. Bar, 200 µm. (B) Average speeds of the wild-type (WT, unlabeled) and *Ate1* knockout (KO, GFP-labeled) cells moving in co-culture show that the difference between the speeds of the two cell types is much less than in individual cultures. Average migration speed of wild-type cells was 24.12+/−4.00(SEM), and the knockout 11.29+/−2.78(SEM), n = 4. (C) Last frame of a 12-hour time lapse series of GFP-labeled wild-type cells co-cultured with *Ate1* knockout cells moving into the wound, overlayed similarly to that shown in (A).

After the scratching of the wound the wild-type cells recovered first; even though areas with GFP-expressing cells at the edge were chosen for observation, shortly after the start of the time lapse, wild-type cells moved to the front and continued leading the way for the entire observation period (Figure 7A and Video S5). *Ate1* knockout cells moved prominently slower than wild-type, however, unlike in individual cultures, co-cultured *Ate1* knockout cells showed a less significant difference in migration speed from the wild-type cells (around 2-fold, Figure 7B) and were able to cover larger distances. This observation suggests that wild-type cells are capable of aiding the knockout cells along to cover larger distances over a period of time. Observations of the time-lapse images (Video S5) suggested that rather than moving, *Ate1* knockout cells, when possible, ‘rode’ on the expanding wild-type monolayer, possibly due to their poorer substrate attachment, and that their ability to cover greater distances was likely due to passive, rather than active movement. Reciprocal assay, with GFP-labeled wild-type cells co-cultured with unlabeled knockout cells, confirmed that, independently of the GFP transfection, wild-type cells indeed migrated faster and filled the wound sooner than the knockout (Figure 7C). It should be noted that in the co-culture assay the wild-type cells on average moved somewhat slower than when cultured individually. A possible explanation for this effect could be that in this assay we did not wait for the edge of the monolayer to recover after the scratch wound and the delay may be simply due to the additional time the dislodged cells at the edge needed to re-attach and polarize.

Thus, *Ate1* knockout in the mesenchymal cells results in greatly delayed migration speeds that originate at the intracellular level and are likely to result in severe morphogenic defects in vivo.

## Discussion

Our data show that knockout of the arginyltransferase *Ate1* in the cells of the neural crest lineage results in multiple morphogenic defects and perinatal lethality in mice. It has been previously shown that complete *Ate1* knockout in mice leads to embryonic lethality and defects in cardiovascular development and angiogenesis [9] that are reminiscent of the defects seen in mouse models with knockout of genes implicated in cell adhesion and migration during embryogenesis [10]. Here we show for the first time that *Ate1* deletion in the migratory subpopulations of the neural crest cells leads to delayed development and reduced size of the neural crest-derived organs and tissues, suggesting that *Ate1*-dependent migration of the neural crest cells is essential for normal embryogenesis.

We have previously shown that *Ate1* knockout embryonic fibroblasts have leading edge defects that arise from abnormalities in the non-arginylated actin cytoskeleton [24]. Here we found that in addition to the abnormal leading edge *Ate1* knockout cells also have impaired adhesion to the extracellular matrix and that the rate of their motility is significantly delayed due to the impaired intracellular machinery rather than to the extracellular environment. It is possible that the defects seen in Wnt1-Ate1 embryos may have additional underlying mechanisms, including impaired epithelio-mesenchimal transformation that could hinder the size of the migratory cell population, or abnormal responses to the signals that coordinate the migratory events on the organismal level. However, the intracellular changes observed in response to *Ate1* knockout are likely sufficient to induce major morphogenic defects even without perturbations in other important developmental processes.

Wnt1-Ate1 mice show defects of varying severity, with a small fraction of mice surviving to adulthood. Our data show that the impairments of neural crest cell migration patterns observed in Wnt1-Ate1 knockout are variable from embryo to embryo, suggesting that local variations in the migration speeds of the *Ate1* knockout cells can contribute to the phenotype variability. Our data on co-culture of wild-type and *Ate1* knockout cells further suggest that *Ate1* knockout cells during migration and tissue expansion can ‘ride’ on the neighboring expanding wild-type tissues, reaching further destinations in the embryo than they could reach on their own. This seems to be especially likely in the case of the enteric neurons that migrate as individual cells on the walls of the expanding gut. Indeed, the migrating speeds of these neurons have been calculated to exceed the average speed of migrating mesenchymal cells in culture and in situ by at least a factor of 2 [10], suggesting that the expanding tissues greatly aid them along in reaching their destination and covering the entire gut wall. Consistent with this fact, Wnt1-Ate1 mice have no defects in gut innervation, while having significant defects in craniofacial migratory neural crest tissues, suggesting that gut neuron precursors in Wnt1-Ate1 knockout reach their destinations mostly by ‘riding’, which is not affected by *Ate1* knockout–related impairments in cell migration speeds.

In addition to the neural crest-derived craniofacial structures, *Wnt1*-expressing cells in the neural tube give rise to the peripheral nervous system and large parts of the brain, including the entire midbrain. In Wnt1-Ate1 mice parts of the peripheral nervous system and the midbrain region are prominently devoid of Ate1 expression (Figure S3 and Figure S4). While we observed no morphological defects in the midbrain or the layout of the peripheral nervous system in the mutant mice, the Wnt1-Ate1 pups that survived for 1–3 weeks past birth exhibited severe behavioral retardation, delayed growth, and apparent difficulty with feeding. While some of these defects, especially the feeding abnormalities, could result from the shortened palates that prevent normal food intake, others could result from the neurological problems, such as reduced innervation leading to the impaired neuromuscular functions and reduced expansion in the thoracic cage (which may result in breathing abnormalities and lung collapse), or from the defects in the sensory organs that are controlled by the midbrain, and possibly from other midbrain-related physiological abnormalities. It has been previously suggested that *Ate1*-dependent arginylation plays an important role in nerve growth and regeneration [17],[47], leading to an interesting possibility that some of the Wnt1-Ate1 mice may have neurological abnormalities of varying severity originated from the developmental defects not directly related to the neural crest cell migration. This possibility requires further study.

Despite prominent *Wnt1* expression in the head and chest area and somites, we saw no visible reduction in *Ate1* staining in such neural crest-derived structures as teeth, endocrine glands, and heart, and no abnormalities were detected in these structures in Wnt1-Ate1 knockout mice. It is possible that in these areas *Wnt1*-expressing cells exist in a mixture with other cell populations, making them difficult to distinguish on tissue sections. In such case, their migration through the embryo could be heavily aided by surrounding tissues and impairment in their migratory machinery would be less visible. It is also possible that these cells normally express lower levels of *Ate1*, making the *Ate1* knockout and the corresponding migratory defects difficult to detect. Further investigation of these differences may lead to important discoveries of the role of arginylation in the development of these organs.

**Figure 8:**
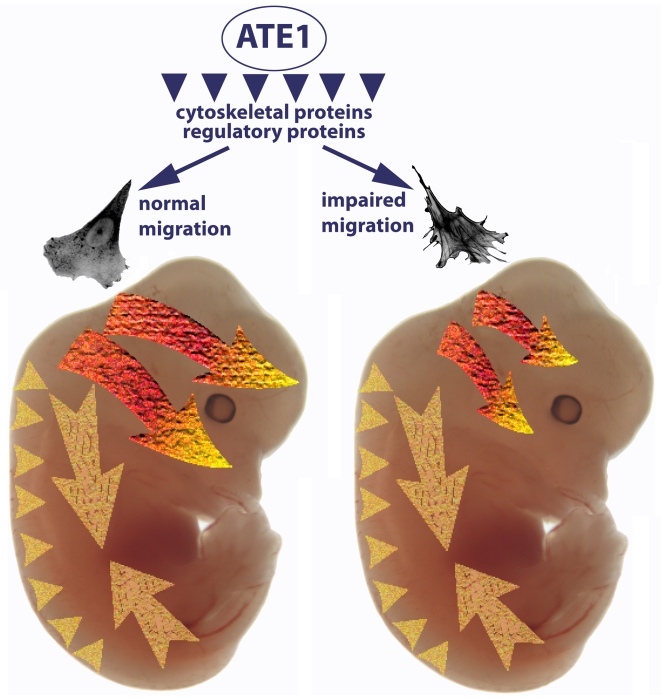
*Ate1*-dependent impairment of cell migration preferentially affects cranial neural crest-dependent morphogenesis. Arginyltransferase *Ate1* has been found to arginylate multiple intracellular targets, including a large subset of cytoskeletal proteins and a smaller subset of regulatory proteins. A combination of these arginylation events results in impairment of cell adhesion and the cytoskeletal structures that lead to slower migration speeds. The three migrating subpopulations of *Wnt1*-expressing cells—cranial neural crest (bright orange-yellow arrows), vagal and sacral neural crest (large pale yellow arrows), and trunk neural crest (pale yellow arrowheads)—are differentially affected by this impaired migration. In the trunk, cells migrating over short distances with relatively slow speeds are apparently capable of getting to their destinations despite the *Ate1*-dependent defects. Cells of the vagal and sacral neural crest migrate individually along the expanding gut; while they are believed to be the fastest migrating neural crest cells, our data suggest that this migration may be greatly aided by the expanding surrounding tissues and that *Ate1*-dependent impairments do not affect the final positioning of these cells in the enteric nervous system. Cranial neural crest giving rise to some of the skull bones (top arrow) and palate (bottom arrow) migrate with steady speeds similar to those observed in culture. Migration of these cells is heavily affected by *Ate1* knockout, resulting in morphogenic defects in multiple craniofacial structures.

Our data show that uniform impairment of the intracellular arginylation-dependent mechanisms of cell migration produces different effects in different *Wnt1*-expressing migratory cell subpopulations during development. These differences may be explained by the diverse mechanisms by which these subpopulations migrate in situ (Figure 8). Cells of the cranial neural crest migrate in large groups from the upper trunk region into the craniofacial areas and contribute to the soft palate, frontal bones, and other craniofacial structures. Vagal and sacral neural crest cells migrate individually along the developing gut from the back and the front, to give rise to the enteric nervous system. Trunk neural crest cells migrate over relatively short distances forming pigment cells, ganglia, and some other structures. Based on the timing of the corresponding developmental events and the distances these cells cover overall, it has been calculated that migration in these subpopulations occurs at different speeds [10], and therefore such migration on the organismal level would be differentially affected by impairment of the same intracellular mechanisms. In the trunk, where cells migrate slowly over short distances, 2–4-fold reduction in the motility rate would be practically unnoticeable on the developmental scale. Consistent with this, we did not observe significant defects in the structures that develop from these cells due to *Ate1* knockout. In the gut, cells migrate individually and while the migration speed of these cells is believed to be the fastest, up until now it has been unknown how much of this speed is contributed by the movement of the expanding tissues rather than the migrating cells themselves. Our data that enteric neurons are positioned normally despite the *Ate1* knockout in these cells points to a possibility that their migration is heavily aided by tissue growth and gut elongation and that the cells themselves do not need to move fast to reach their final position. Cells contributing to craniofacial skeleton migrate as large subpopulations, with predicted speeds close to those experimentally observed for neural crest and other mesenchymal cells in culture and in situ (∼40 µm/hour, see [10] for review). Our data suggest that the migration of these cells depends heavily on the intracellular mechanical components that drive cell adhesion and movement, and that this cell population is affected the most by the impairment in these mechanisms. This is consistent with the results of other studies, where mouse knockouts of the cell migration-related genes (such as fibronectin, cell adhesion molecules, etc., see [10] for review) leads to similar defects.

Recent data suggest that many proteins involved in different physiological events are arginylated [23] and the list of identified arginylation targets is by far not complete. A prominent subset of the identified arginylated proteins (such as actin, etc.) are directly implicated in cell migration and it is very easy to suggest how lack of arginylation could lead to the defects in these proteins and affect cell migration by impairing the intracellular cytoskeleton-related machinery. For example, talin, a major focal adhesion protein, has been found to be arginylated on Ala1903, and the present study suggests that talin-containing focal adhesions are significantly reduced in *Ate1* knockout, pointing to a possibility that talin arginylation is one of the key players in the *Ate1* regulation of cell adhesion. Studies of the regulation of these, and other proteins by arginylation and its contribution to *Ate1*-dependent cell migration constitute exciting directions of further research.

## Materials and Methods

### Transgenic mice

Mice with the exons 1–3 of the *Ate1* gene flanked by LoxP sites (*Ate1*-floxed mice) were generated by introducing a targeting construct into the corresponding genome region in a cassette containing the floxed allele of the *Ate1* genomic region fused with an frt-flanked Neo gene. The targeting vector was constructed using recombineering technique as described in [48]. A 12,375 bp genomic DNA fragment (position Chr 7: 130,302,694–130,315,068 Mouse Feb 2006 Assembly) containing exon 1–4 of the gene was retrieved from BAC clone RP23-92D13. A loxP sequence was inserted 657 bp upstream of exon 1 and A frt-neo-frt-loxP cassette was inserted into the intron 3, 505 bp downstream of exon 3. Thus a fragment of 5,019 bp genomic DNA containing exons 1–3 of the *Ate1* gene was floxed (see Figure S1A). For ES cell targeting, the targeting vector was linearized with Not1 and electroporated into D1 ES cells derived from F1 hybrid blastocysts of 129S6 x C57BL/6J by Gene Targeting & Transgenic Facility at University of Connecticut Health Center. 192 G418 resistant ES colonies were isolated, and 32 clones were screened by nested PCR using primers outside the construct paired with primers inside the neo cassette. The sequences for primers used for ES cell screening were as follows:

5′ arm forward primers: ATE Scr F1 (5′- GTCTCACTTCCCTTCCTTAG -3′) and ATE Scr F2 (5′- ATTACCAGTGCTCGGTGCTT -3′). Reverse primers: Loxp scrR1 (5′–GAGGGACCTAATAACTTCGT-3′) and loxp scrR2 (5′-GGAATTGGGCTGCAGGAATT-3′). 3′ arm forward primers: frt scr F1 (5′-TTCTGAGGCGGAAAGAACCA-3′) and frt scr F2, (5′-CGAAGTTATTAGGTGGATCC-3′); Reverse primers: ATE Scr R1 (5′- tcagtggttctcaacctgtg -3′) and ATE Scr R2 (5′- caggggttacctaagaccat -3′);

7 out of 32 clones were PCR positive for both arms and were expanded. The genotypes were confirmed after ES cell expansion.

For chimera generation and F1 mice genotype analysis three clones (1B3, 1A4 and 1H2) were aggregated with 8-cell embryos of CD-1 strain. The aggregated embryos were transferred to pseudopregnant recipients and allowed to develop to term. 25 chimeric mice were identified by coat color. Five chimeras (2 each for1B3 and 1A4, 1 for 1H2) were mated with CD-1 females to test for germline transmission. All of them were demonstrated as germline chimeras. The neo cassette was removed by mating the chimeras with ROSA26FLP1 (Jax stock#: 003946) homozygous females.

To obtain Wnt1-Ate1 mice, *Ate1*-floxed mice were crossed with the mouse strain Tg(*Wnt1*-GAL4)11Rth Tg(*Wnt1*-cre)11Rth/J (The Jackson Laboratory) expressing Cre recombinase under the neural crest-inducing *Wnt1* promoter. The following primers were used for genotyping of the final mouse strain (see Figure S1B for a typical genotyping gel): for *Ate1*-floxed allele, *Ate1*gtLoxF (5′-TGCCTCCAGCATTGGATGAA-3′) and *Ate1*gtLoxR (5′-CCATGGGTCTCCAATTTGCA-3′); For ROSA locus and Wnt transgene, primers recommended by the Jackson Laboratory were used as described on their web site for each corresponding strain.

### Ate1 antibody

Rat monoclonal antibodies against a mixture of full-length bacterially expressed Ate1-1 and Ate1-2 were custom produced by Absea (http://www.absea-antibody.com). Reactivity with individual Ate1 isoforms was verified by Western blots against purified bacterially expressed Ate1, isoforms 1–4. Clone 6F11 that interacts equally with all four Ate1 isoforms was used for section staining shown in Figure S3 and Figure S4.

### Anatomical and histological analysis

For gross anatomical examination, mice at different postnatal stages were euthanized, dissected and observed for possible organ and tissue abnormalities, and individual organs were collected and compared between the mutants and the matching controls. For obtaining the images of the roof of the mouth and nasopharynx entrance shown in Figure 2 newborn mice were fixed in 4% paraformaldehyde in PBS and stored at 4°C. Fixed samples were washed in PBS and lower jaws were removed, and then the palate and the entrance of nasopharynx were observed under a dissection microscope.

For histological analysis, mice and isolated mouse organs including X-gal stained embryos were fixed in 4% paraformaldehyde in PBS, paraffin embedded, and sectioned. For observation and analysis of general organ morphology sections were stained with hematoxylin and eosin. For immunohistochemistry shown in Figure S3 and Figure S4, paraffin-embedded sections were deparaffinized with xylene, re-hydrated with sequential methanol∶PBS series (95∶5, 70∶30, 50∶50 and 30∶70), washed with PBS, blocked with PBS supplemented with 0.1% Triton X-100 and BSA, and treated with anti-*Ate1* (described above, 1∶50), anti-beta-III tubulin (R&D Systems, MAB1195, 1∶100), anti-phospho histone H3 (Santa Cruz Biotechnology, sc-8656, 1∶500), anti-cleaved caspase 3 (Cell Signaling Technology, #9661, 1∶100) or anti-talin (Santa Cruz Biotechnology, sc-7534, 1∶500) antibodies in PBS supplemented with 0.1% Triton X-100. After washing with 0.1% Triton X-100 in PBS, samples were treated with fluorescent dye-conjugated secondary antibodies, washed with PBS and mounted in Aqua Poly/Mount (Polysciences, Inc., 18606). Samples were observed under a fluorescent microscope.

### Whole-mount skeletal staining

Whole-mount skeletons of newborn and adult mice were stained with alizarin red S and alcian blue 8GS as described in [41].

### Whole-mount immunostaining for enteric nerves

Fixation and blocking in guts excised from embryos at E16.5 was performed as described in [49]. After blocking, guts were incubated with anti beta-III tubulin antibody for 2 days at 1∶100 dilution in 5% BSA in TST buffer (20 mM Tris-HCl (pH 8.0), 150 mM NaCl, 0.1% Triton X-100), washed with TST, and treated with FITC-conjugated secondary antibody at 1∶100 dilution in TST. After washing in TS buffer (20 mM Tris-HCl (pH 8.0), 150 mM NaCl), samples were mounted in OxyFluor at 1∶100 dilution in TS, and observed under confocal microscope.

### Whole-mount in situ hybridization

Riboprobe generation and in situ hybridization were performed according to [50] with a modification in post-antibody wash as follows: after the treatment with the antibody, embryos were washed 6 times for 1 hour each and stored at 4°C overnight. This washing process was done 3 times over the course of 3 days. After color development, embryos were fixed, dehydrated, rehydrated and cleared according to the protocol available online (http://www.med.upenn.edu/mcrc/histology_core/wholemount.shtml).

### Cultures of neural crest explants

Embryos at E8.5 or E9.5 were collected from pregnant female mice into PBS, and neural tube area was dissected from the back of the embryos using 27 gauge needles. Dissected tissues were cultured in 1∶1 of DMEM/F10 supplemented with 10% FCS and antibiotics on glass bottom dishes (Matek) pre-coated with 10 µg/ml fibronectin in PBS for 1 hour at room temperature. Explants were cultured for 2 days and used for further analyses.

### Cell migration assays

Immortalized wild-type and *Ate1* KO embryonic fibroblasts were cultured as described in [24]. Wound healing assays to measure the migration speeds of these cells were performed by growing the cells to a confluent monolayer in tissue culture dishes, followed by scraping off a portion of the monolayer, and the migration of the cells into the resulting wound was recorded as time-lapse images at 2 min intervals over 10–15 hour observation period and analyzed using Metamorph imaging software (Molecular Devices).

To analyze the migration of *Ate1* KO cells in co-culture with wild-type cells, wound healing experiment were performed with GFP-labeled *Ate1* KO cells co-plated with wild-type cells as a mixture at the ratio of KO∶WT 1∶2 to 1∶6. For reciprocal experiments, GFP-labeled wild-type and unlabeled *Ate1* KO cells were used.

### Immunocytochemistry in cultured cells

For immunostaining, WT and *Ate1* KO cell monolayers were scraped to produce the wound. After 4 hours, cells were fixed with 4% paraformaldehyde, permeabilized with 1% Triton X-100, and stained with mouse monoclonal anti-paxillin antibody (BD Biosciences), rhodamine-phalloidin (Sigma), mouse anti-talin clone 8d4 (Sigma), and mouse anti-FAK, clone 2A7 (Upstate Biotechnology) in different combinations. For immunostaining for fibronectin, cells attached on the cover glasses were fixed in 4% paraformaldehyde and stained with rabbit polyclonal anti-fibronectin antibody (Sigma). To observe only the extracellular fibronectin, cells were not permeabilized or washed with Triton X-100 before or after fixation.

### X-gal staining in fetuses and neural crest explants

Fetuses or neural crest explants were fixed with 4% paraformaldehyde at 4C for 1 hour, rinsed for 30 minutes with rinse buffer (0.2 M sodium phosphate pH 7.3, 2 mM magnesium chloride, 0.02% IGEPAL and 0.01% sodium deoxycholate) 3 times, and incubated overnight at 37°C in the staining solution (5 mM potassium ferricyanide, 5 mM potassium ferrocyanide and 1 mg/ml X-gal in rinse buffer). Samples were post-fixed with 4% paraformaldehyde and stored in 70% ethanol.

### Detection of apoptosis in fetuses by TUNEL assay

TUNEL assay were performed in fetuses at E9.5 and E12.5 using In Situ Cell Death Detection Kit, Fluorescein (Roche). Fetuses were fixed in 4% paraformaldehyde in PBS overnight at 4°C, washed 3 times in PBS and incubated with 18.7 µg/ml proteinase K in 10 mM Tris/HCl, pH 7.5 for 30 minutes at 37°C. After washing 3 times in PBS, samples were incubated in the Label Solution supplemented with Enzyme Solution for 1 hour at 37°C, then washed in PBS 3 times and photographed under a fluorescent microscope. For negative control, samples were incubated in Label Solution without Enzyme Solution. For positive control, samples were treated with 100 U/ml DNase I and 1 mg/ml BSA in 50 mM Tris/HCl, pH 7.5 for 20 minutes at room temperature, followed by incubation with Label Solution supplemented with Enzyme Solution.

### Detection of BrdU incorporation in neural crest explants by immunostaining

Cell proliferation in the neural crest explants from E8.5 and E9.5 fetuses was analyzed by detecting BrdU incorporation using BrdU Cell Proliferation Assay kit (Calbiochem, QIA58). After 2-day culture, explants were incubated with 1∶2000 BrdU Label in culture medium for 2 hours. Explants were washed twice in DMEM without serum, and fixed with Fixative/Denaturing solution at room temperature for 30minutes. After washing in 70% ethanol, samples were air-dried and incubated with 1∶100 Anti-BrdU antibody in Antibody Diluent at room temperature for 1 hour. Samples were washed twice in Wash Buffer (1∶20 Plate Wash Concentrate in water), twice in PBS, and treated with 1∶200 Cy3 conjugated secondary antibody (Jackson ImmunoResearch Laboratories) in PBS at room temperature for 1 hour. Samples were washed 3 times in PBS and photographed under a fluorescent microscope.

## Supporting Information

Figure S1 (A) Construction of the *Ate1* conditional knockout and Wnt1-Ate1 mice. (B) Genotyping of *Ate1*-floxed and Wnt1-Ate1 mice.
(0.76 MB TIF)

Figure S2 Ate1 antibody characterization. Left, Coomassie-stained gel of wild-type and knockout cell extracts. Right top, immunoblots of the cell extracts shown on the left. Ate1 antibody specifically recognizes a ∼55 kDa band in the wild-type but not knockout extract. Right bottom, immunoblots of bacterially expressed Ate1 isoforms show that Ate1 antibody reacts equally with all four isoforms.
(3.06 MB TIF)

Figure S3 *Ate1* deletion in Wnt1-Ate1 mice in midbrain and enteric neurons. (A) An H&E-stained sagittal section of an embryo at E16.5. Large and small box outline the areas of the animal corresponding to those shown in (B,C), respectively. (B) Midbrain area of the control (WT) and Wnt1-Ate1 (KO) newborn mouse double-stained for the neuronal marker beta-III tubulin and *Ate1*. In KO, *Ate1* is prominently missing from the midbrain, but not from other areas of the embryo. (C) Cross sections of the gut in control (WT) and Wnt1-Ate1 (KO) newborn co-stained for *Ate1* and beta-III tubulin show prominent absence of *Ate1* from the enteric neurons. Decrease in the *Ate1* signal level compared to the control was similar in the gut neurons and in mid-brain, as verified by measurement of the fluorescence levels.
(3.25 MB TIF)

Figure S4 *Ate1* deletion in Wnt1-Ate1 mice affects some but not all of the peripheral nervous system. Midbrain (m) and hindbrain (h) area of the control (WT) and Wnt1-Ate1 (KO) E12.5 embryo double-stained for the neuronal marker beta-III tubulin and *Ate1*. *Ate1* is prominently missing from the midbrain and some peripheral nervous system structures (arrowheads).
(3.87 MB TIF)

Figure S5 Enteric neurons are positioned normally in Wnt1-Ate1 mice. Mid-areas of whole mount E16.5 guts stained for beta-III tubulin.
(2.44 MB TIF)

Figure S6 Impaired cell migration in Wnt1-Ate1-R26R neural crest explants. X-gal staining of control (left four panels) and Wnt1-Ate1 (right four panels) explants derived from E8.5 embryos show that while LacZ-expressing cells in control actively emigrate from the explant and reach the periphery of the expanding cell mass, cells in the mutant stay closer to the explant and do not appear to venture out on their own. 10× images on the periphery show higher magnifications of the regions boxed in the 2× images of the corresponding explants in the center, illustrating the cell emigration from the explant mass in control (left) and absence of such emigration in the knockout (right). 10 control and 6 mutant explants were analyzed.
(4.60 MB TIF)

Figure S7 *Ate1* knockout in Wnt1-Ate1 embryos does not result in increased rates of apoptosis. Left, lower magnification image of a control DNaseI-treated embryo stained with TUNEL. Boxed regions outline the areas of the head (1), pharyngeal arches (2), and back (3), shown magnified in the right-hand panels for TUNEL-stained wild-type, Wnt1-Ate1, negative, and positive control embryos as marked. Levels of TUNEL staining in Wnt1-Ate1 and wild-type embryos are similar to those in the negative control and do not show any prominent differences from each other. 4 wild-type and 2 Wnt1-Ate1 embryos were analyzed.
(1.82 MB TIF)

Figure S8 *Ate1* knockout in Wnt1-Ate1 embryos does not result in increased rates of apoptosis. Cross sections of wild-type (top) and Wnt1-Ate1 (bottom) embryos at E9.5 stained with an antibody to cleaved caspase 3. The area shown includes neural tube with adjacent population of migratory neural crest cells (see Figure S13C for X-gal staining of a similar section). Levels of cleaved caspase 3 staining in Wnt1-Ate1 and wild-type embryos do not show any prominent differences from each other and from the negative control with secondary antibody only.
(1.22 MB TIF)

Figure S9 *Ate1* knockout does not affect neural crest cell proliferation rates. X-gal stained E9.5 wild-type (top) and Wnt1-Ate1 (bottom) embryos were sectioned and immunostained for cell proliferation marker phospho-histone H3. No differences in staining were observed between wild-type and Wnt1-Ate1 in X-gal stained tissues.
(4.03 MB TIF)

Figure S10 *Ate1* knockout does not affect neural crest cell proliferation rates. Neural crest explants from E8.5 and E9.5 *Ate1* knockout embryos labeled with BrdU after 2 days in culture. Levels of BrdU staining are similar in wild-type and knockout explants, suggesting no differences in proliferation rates of the neural crest cells.
(3.57 MB TIF)

Figure S11 *Ate1* knockout results in reduced focal adhesions. Left panels, fluorescence staining of the edge of the cell monolayer moving into the wound with anti-paxillin (top), anti-talin (middle), and anti-focal adhesion kinase (FAK, bottom) to visualize focal adhesions. Knockout cells show a dramatic reduction in focal adhesion area and number. Bar, 20 µm. Middle panels, Western Blotting comparison of the focal adhesion protein levels in wild-type and *Ate1* knockout cells. Loading was adjusted by weight of the packed cell pellets and verified by loading control–actin for paxillin, and tubulin for talin and FAK, as shown in the image. Right panels, quantification of the number of paxillin focal adhesions per µm of the wound edge and of the area of talin focal adhesions per cell leading edge shows that the number of prominent focal adhesions in wild-type exceeds that in the knockout by over 5-fold. Error bars for paxillin represent SEM for the measurements in 21 and 18 different images in WT and KO, respectively. Error bars for talin represent SEM for the measurements of 15 WT and 18 KO cells.
(2.18 MB TIF)

Figure S12 *Ate1* knockout results in reduced focal adhesions. Left panels, fluorescence staining of single cells with anti-talin (top) and anti-paxillin (bottom) to visualize focal adhesions. Knockout cells show a dramatic reduction in focal adhesion area and number. Right panels, quantification of the number of focal adhesion area per cell shows that the number of prominent focal adhesions in wild-type exceeds that in the knockout by several fold. Quantifications shown represent measurements of 15 WT and 15 KO cells (paxillin) and 21 WT and 25 KO cells (talin).
(2.07 MB TIF)

Figure S13 *Ate1* knockout results in reduced focal adhesions. Cross sections of wild-type (A) and Wnt1-Ate1 (B) embryos at E9.5 stained with antibody to talin. C, a similar area from an X-gal stained embryo showing the location of the migratory neural crest cell population used for the quantification of talin levels shown in D. Error bars represent SEM for measurements of 6 regions taken from each of the 5 wild-type and 4 Wnt1-Ate1 sections (30 wild-type and 24 Wnt1-Ate1 measurements), p-value<0.01.
(2.98 MB TIF)

Figure S14 Images of X-gal-stained E9.5 wild-type (left) and Wnt1-Ate1 (right) embryos used for obtaining higher magnification views shown in Figure 4 of the main text. Embryos were staged by somite count, indicated for each embryo on the top right next to the head. For some embryos (marked with X and asterisks) somite counts were not performed. These embryos were staged by comparison with their littermates as follows: *–littermates (shown on right) had 25 and 28 somites; **–littermate (shown on left) had 21 somites; ***–littermates had 22–26 somites; X–four embryos from the same litter lined up on the bottom for comparison.
(4.29 MB TIF)

Figure S15 Images of *Sox10*-stained E9.5 and E10.5 wild-type and Wnt1-Ate1 embryos used for obtaining higher magnification views shown in Figure 4 of the main text.
(4.99 MB TIF)

Video S1 Newborn Wnt1-Ate1 pup (left bottom) exhibits shallow, rapid breathing, eventually resulting in the accumulation of air in the abdominal cavity and death (see Figure 1 in the main text). In contrast its littermate control (right top) breathes regularly and exhibits no visible abnormalities.
(4.61 MB AVI)

Video S2 Four littermate mice at P9 show different behavior depending on the genotype. While control mice (two larger pups) move actively, Wnt1-Ate1 mice (two smaller pups, one on right and one hidden under the other pups at the beginning of the video) don't move around much an appear to be ‘frozen’ in place. Such mice die within the first three weeks after birth (see Table 1).
(3.20 MB AVI)

Video S3 Wild-type cells moving into the wound cover the entire wound area over 12 hours.
(2.69 MB AVI)

Video S4 *Ate1* knockout cells moving into the wound are significantly slowed down and do not cover large areas over extended periods of time.
(2.70 MB AVI)

Video S5 *Ate1* knockout cells (red) co-cultured with wild-type are able to cover larger distances than when migrating by themselves. Some knockout cells could be clearly seen ‘riding’ on the migrating wild-type cells rather than moving on their own.
(1.78 MB AVI)
