# Supplementary figures and images for: Arginylation-Dependent Neural Crest Cell Migration Is Essential for Mouse Development
Source: PLoS Genet. 2010 Mar 12;6(3):e1000878. doi: 10.1371/journal.pgen.1000878 (PMC2837401; doi:10.1371/journal.pgen.1000878)

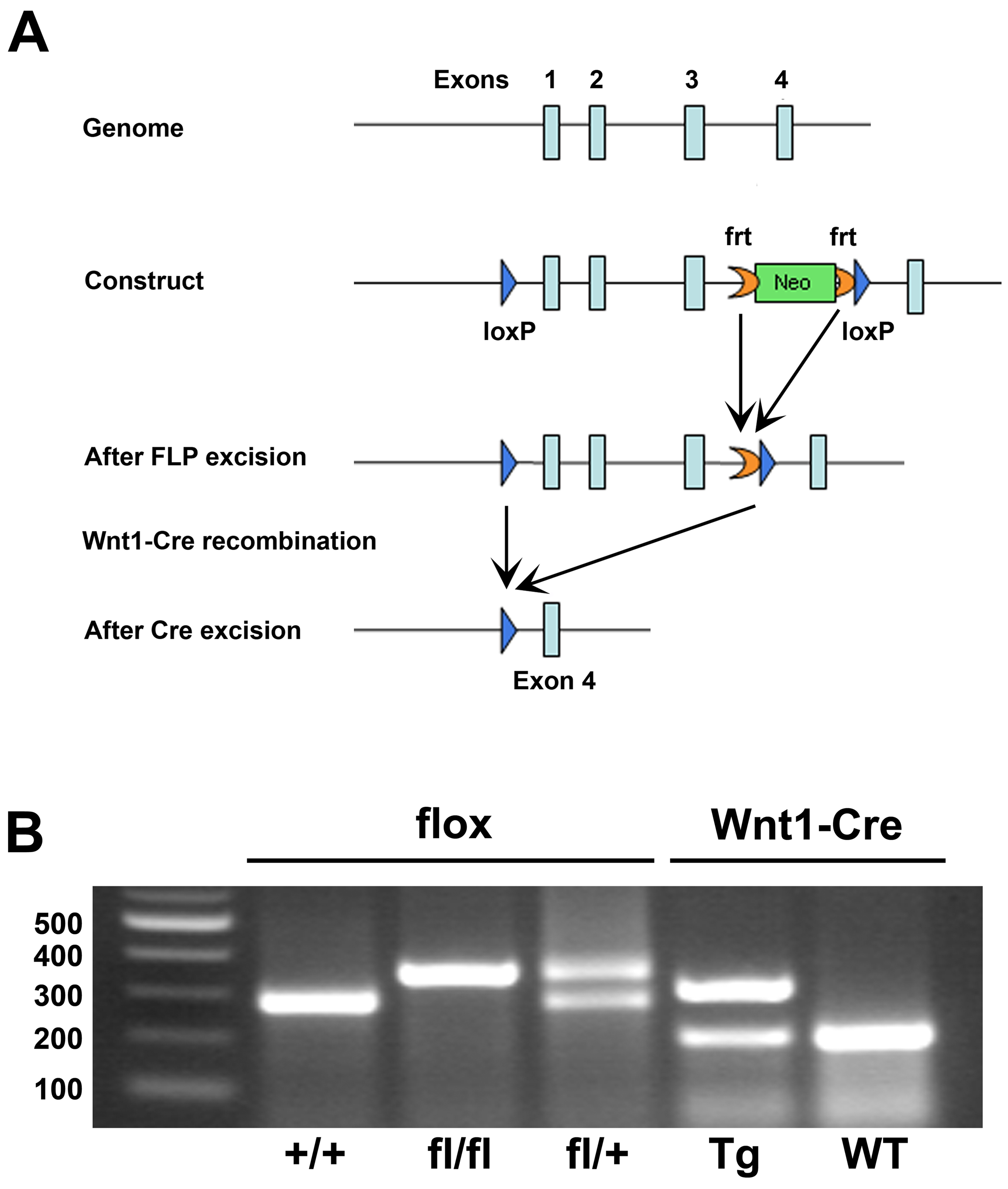

Supplement: Figure S1 — (A) Construction of the Ate1 conditional knockout and Wnt1-Ate1 mice. (B) Genotyping of Ate1-floxed and Wnt1-Ate1 mice. (0.76 MB TIF) [file pgen.1000878.s001.tif]

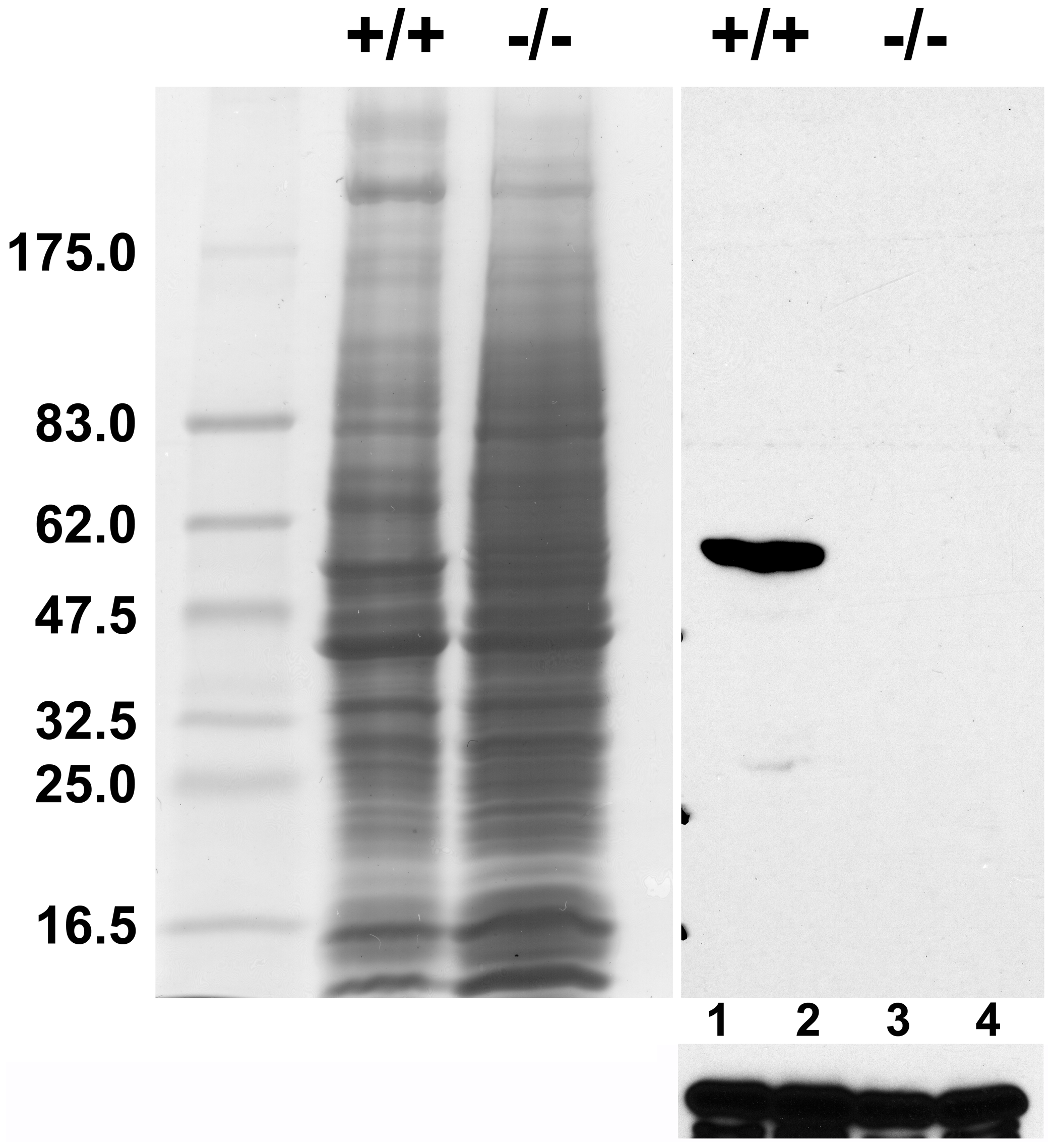

Supplement: Figure S2 — Ate1 antibody characterization. Left, Coomassie-stained gel of wild-type and knockout cell extracts. Right top, immunoblots of the cell extracts shown on the left. Ate1 antibody specifically recognizes a ∼55 kDa band in the wild-type but not knockout extract. Right bottom, immunoblots of bacterially expressed Ate1 isoforms show that Ate1 antibody reacts equally with all four isoforms. (3.06 MB TIF) [file pgen.1000878.s002.tif]

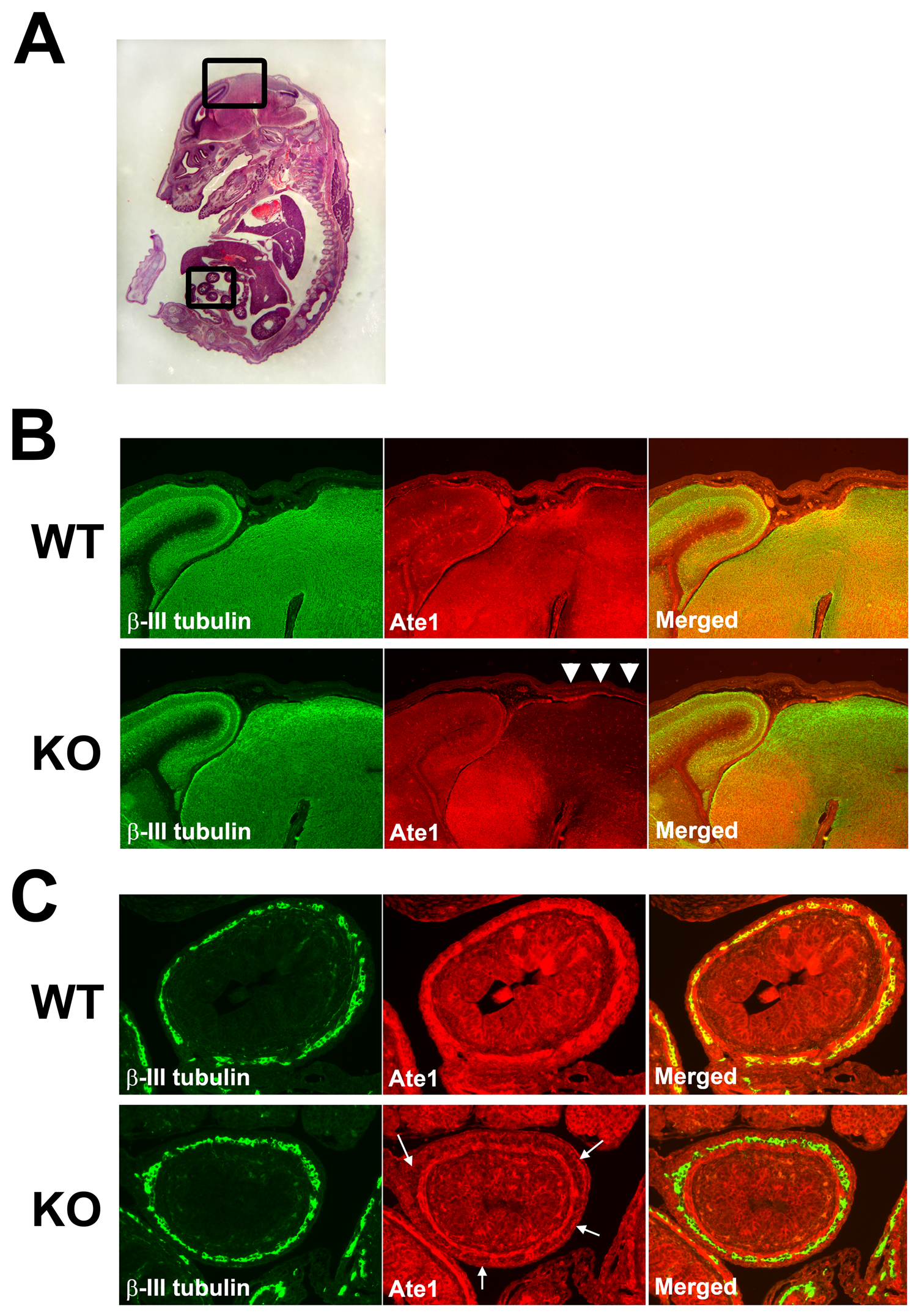

Supplement: Figure S3 — Ate1 deletion in Wnt1-Ate1 mice in midbrain and enteric neurons. (A) An H&E-stained sagittal section of an embryo at E16.5. Large and small box outline the areas of the animal corresponding to those shown in (B,C), respectively. (B) Midbrain area of the control (WT) and Wnt1-Ate1 (KO) newborn mouse double-stained for the neuronal marker beta-III tubulin and Ate1. In KO, Ate1 is prominently missing from the midbrain, but not from other areas of the embryo. (C) Cross sections of the gut in control (WT) and Wnt1-Ate1 (KO) newborn co-stained for Ate1 and beta-III tubulin show prominent absence of Ate1 from the enteric neurons. Decrease in the Ate1 signal level compared to the control was similar in the gut neurons and in mid-brain, as verified by measurement of the fluorescence levels. (3.25 MB TIF) [file pgen.1000878.s003.tif]

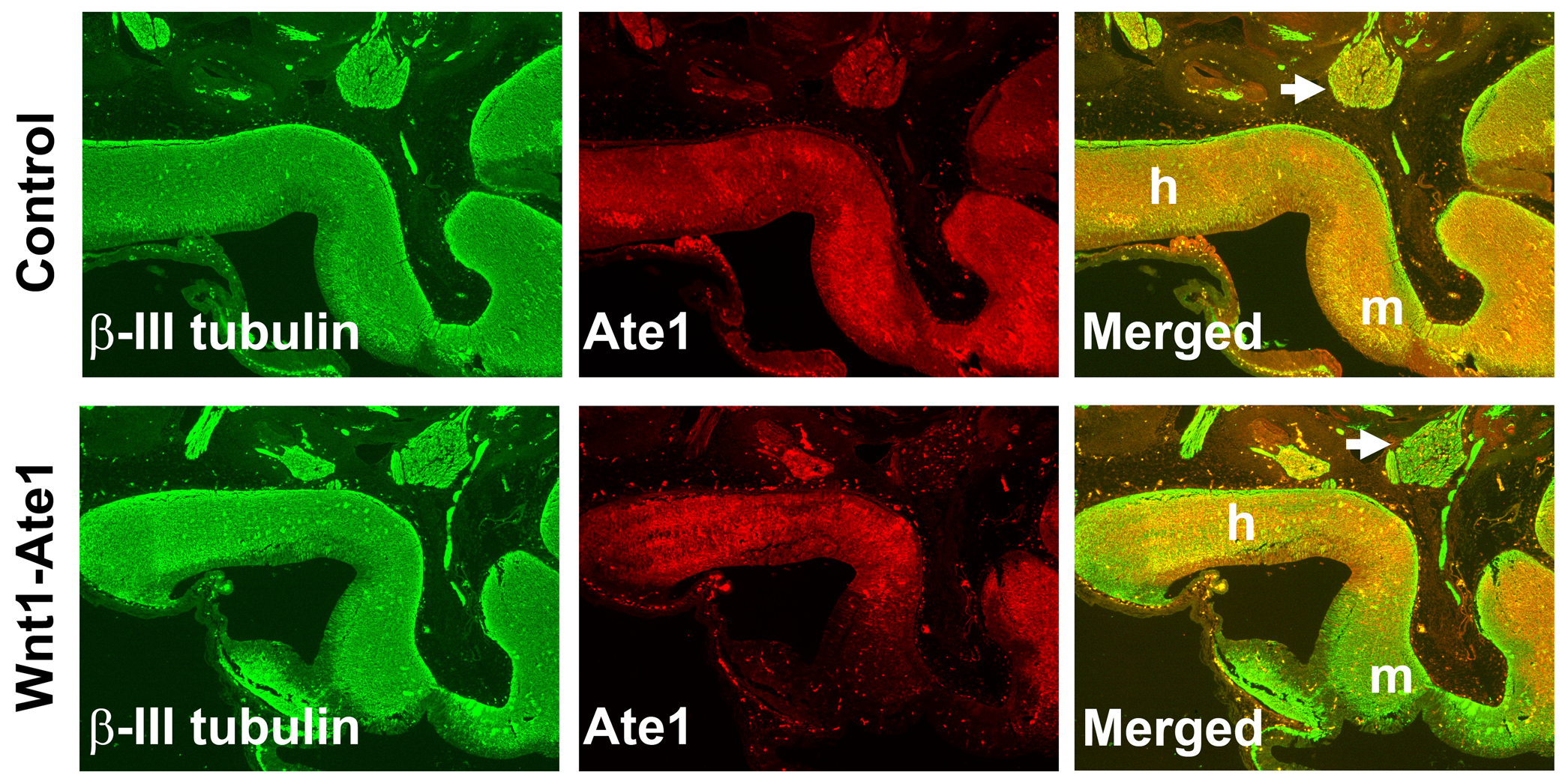

Supplement: Figure S4 — Ate1 deletion in Wnt1-Ate1 mice affects some but not all of the peripheral nervous system. Midbrain (m) and hindbrain (h) area of the control (WT) and Wnt1-Ate1 (KO) E12.5 embryo double-stained for the neuronal marker beta-III tubulin and Ate1. Ate1 is prominently missing from the midbrain and some peripheral nervous system structures (arrowheads). (3.87 MB TIF) [file pgen.1000878.s004.tif]

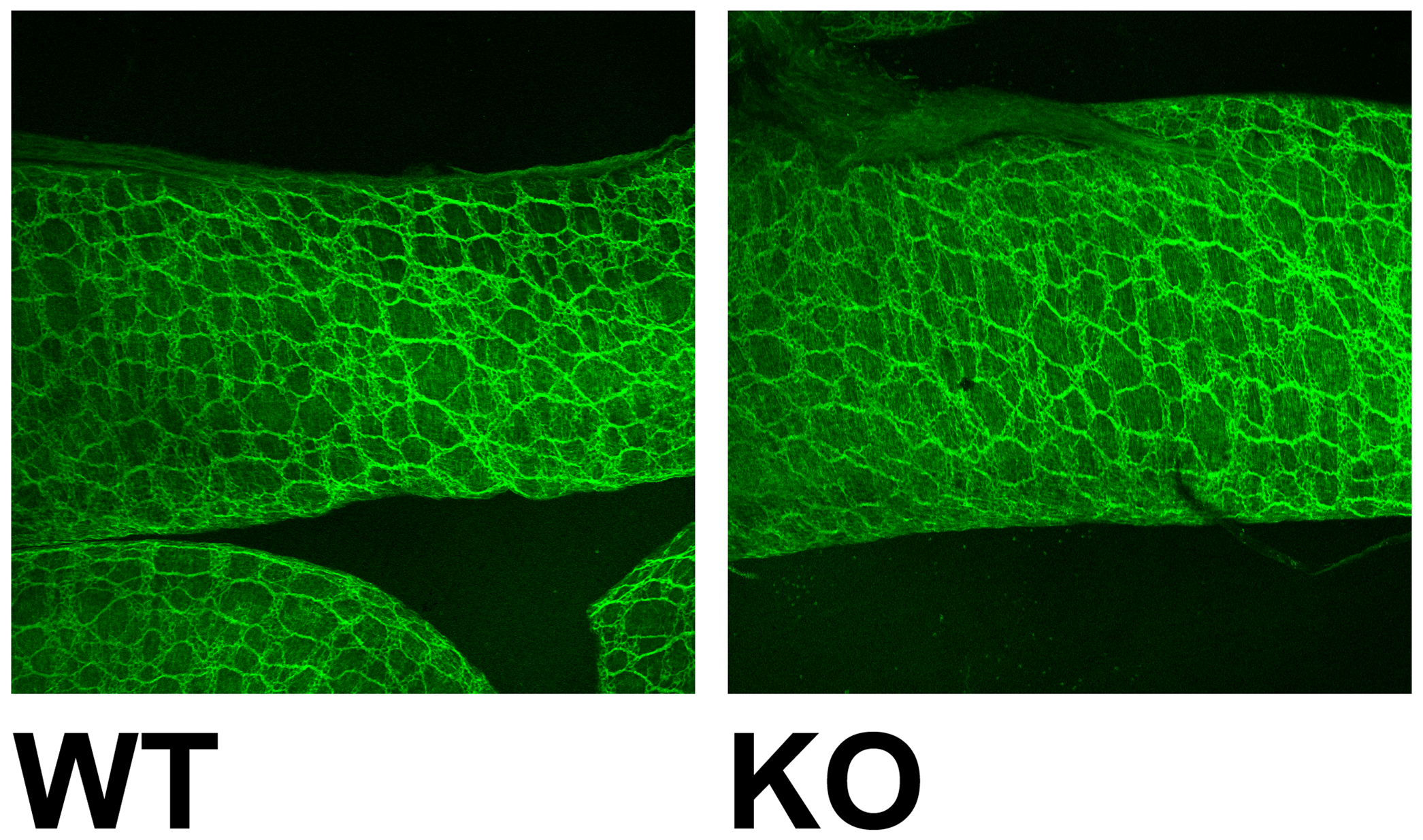

Supplement: Figure S5 — Enteric neurons are positioned normally in Wnt1-Ate1 mice. Mid-areas of whole mount E16.5 guts stained for beta-III tubulin. (2.44 MB TIF) [file pgen.1000878.s005.tif]

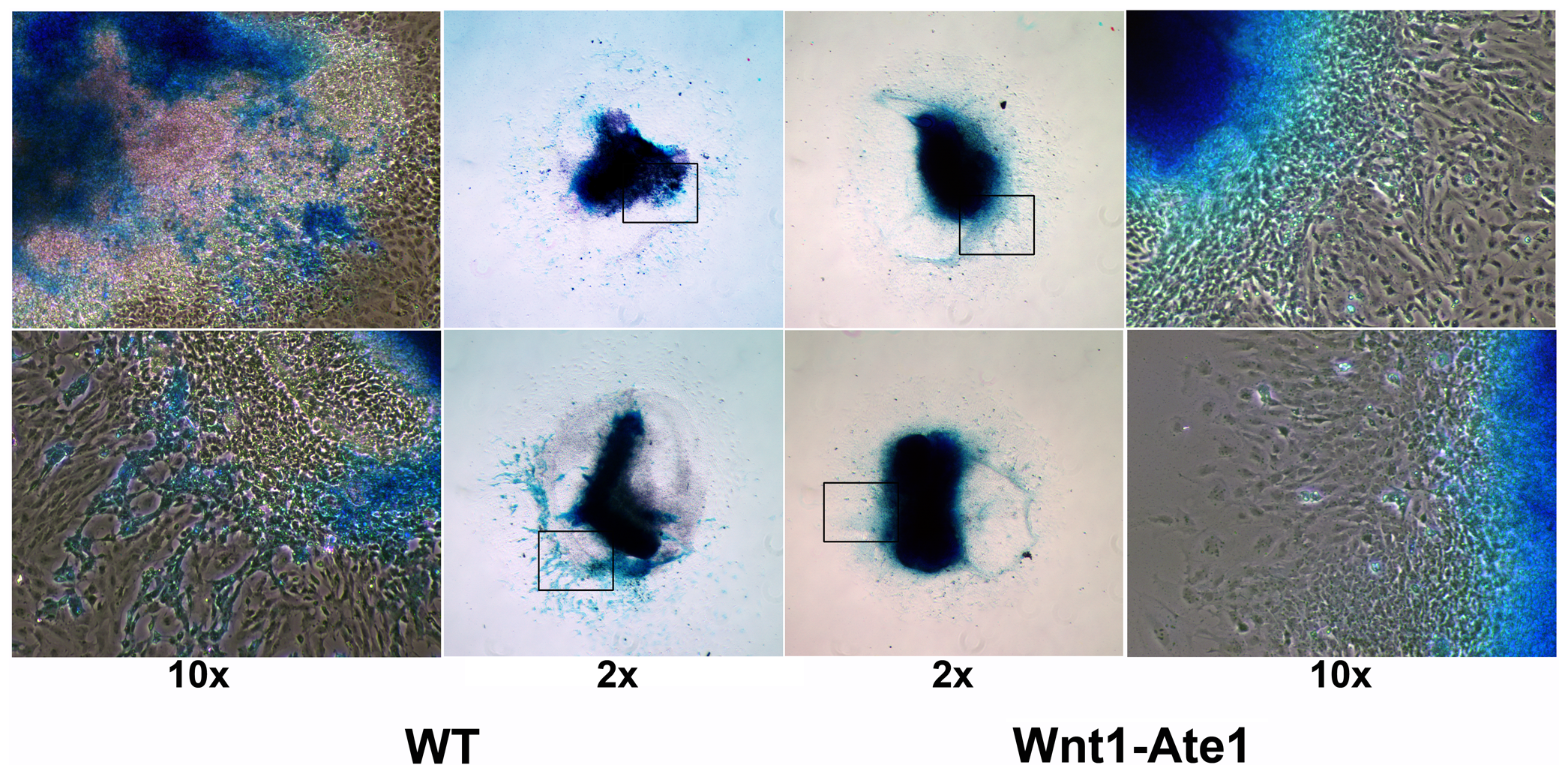

Supplement: Figure S6 — Impaired cell migration in Wnt1-Ate1-R26R neural crest explants. X-gal staining of control (left four panels) and Wnt1-Ate1 (right four panels) explants derived from E8.5 embryos show that while LacZ-expressing cells in control actively emigrate from the explant and reach the periphery of the expanding cell mass, cells in the mutant stay closer to the explant and do not appear to venture out on their own. 10× images on the periphery show higher magnifications of the regions boxed in the 2× images of the corresponding explants in the center, illustrating the cell emigration from the explant mass in control (left) and absence of such emigration in the knockout (right). 10 control and 6 mutant explants were analyzed. (4.60 MB TIF) [file pgen.1000878.s006.tif]

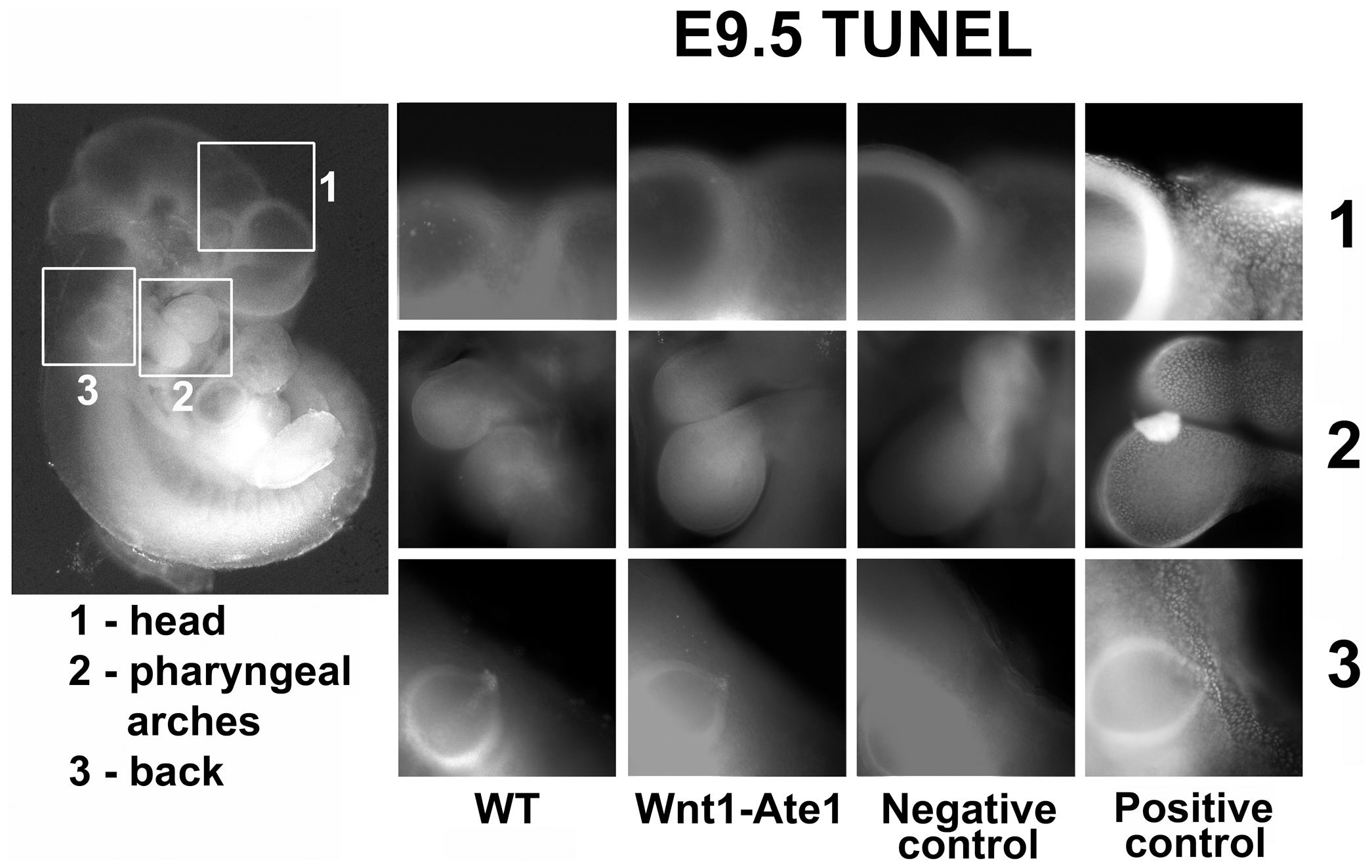

Supplement: Figure S7 — Ate1 knockout in Wnt1-Ate1 embryos does not result in increased rates of apoptosis. Left, lower magnification image of a control DNaseI-treated embryo stained with TUNEL. Boxed regions outline the areas of the head (1), pharyngeal arches (2), and back (3), shown magnified in the right-hand panels for TUNEL-stained wild-type, Wnt1-Ate1, negative, and positive control embryos as marked. Levels of TUNEL staining in Wnt1-Ate1 and wild-type embryos are similar to those in the negative control and do not show any prominent differences from each other. 4 wild-type and 2 Wnt1-Ate1 embryos were analyzed. (1.82 MB TIF) [file pgen.1000878.s007.tif]

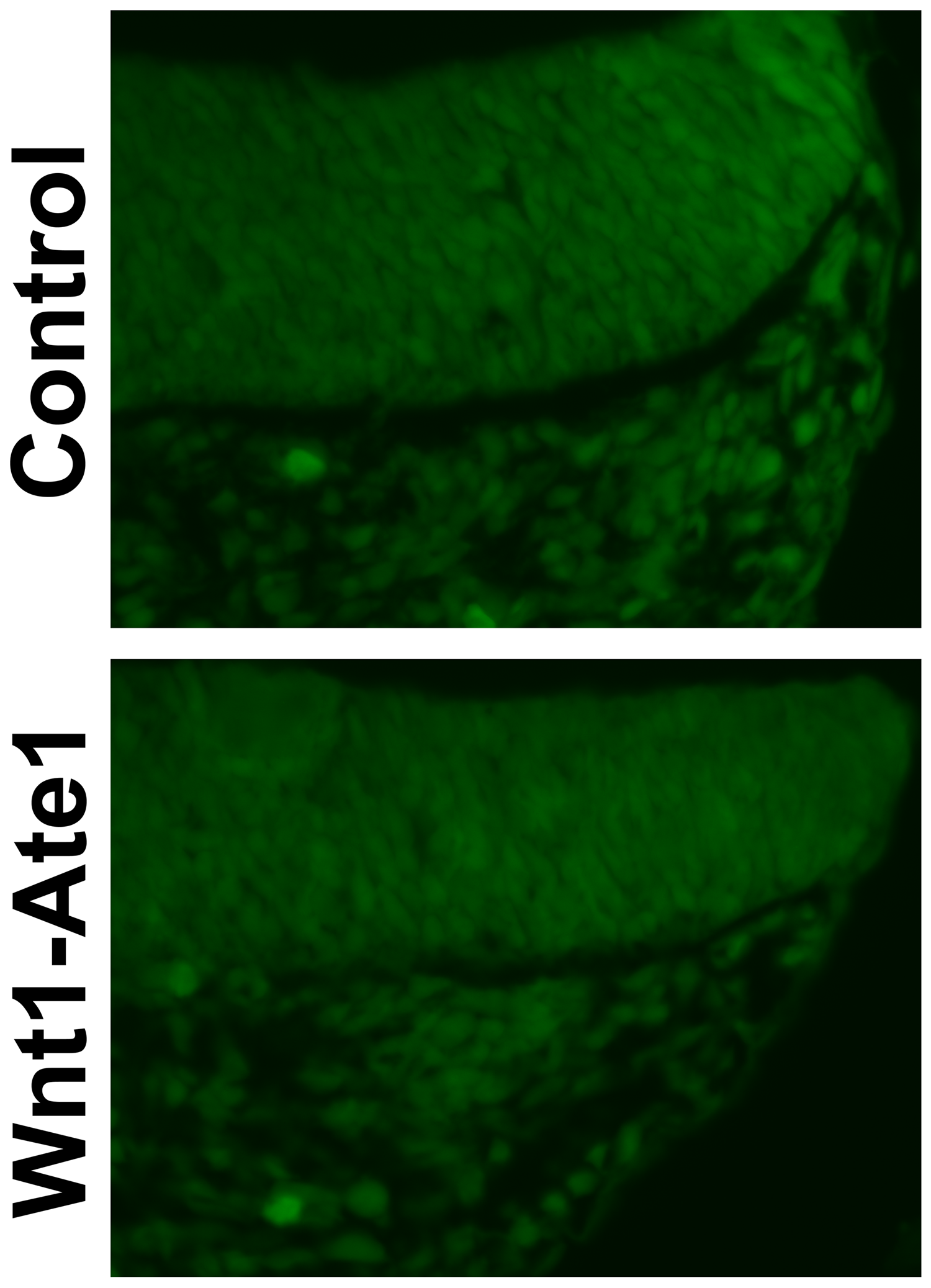

Supplement: Figure S8 — Ate1 knockout in Wnt1-Ate1 embryos does not result in increased rates of apoptosis. Cross sections of wild-type (top) and Wnt1-Ate1 (bottom) embryos at E9.5 stained with an antibody to cleaved caspase 3. The area shown includes neural tube with adjacent population of migratory neural crest cells (see Figure S13C for X-gal staining of a similar section). Levels of cleaved caspase 3 staining in Wnt1-Ate1 and wild-type embryos do not show any prominent differences from each other and from the negative control with secondary antibody only. (1.22 MB TIF) [file pgen.1000878.s008.tif]

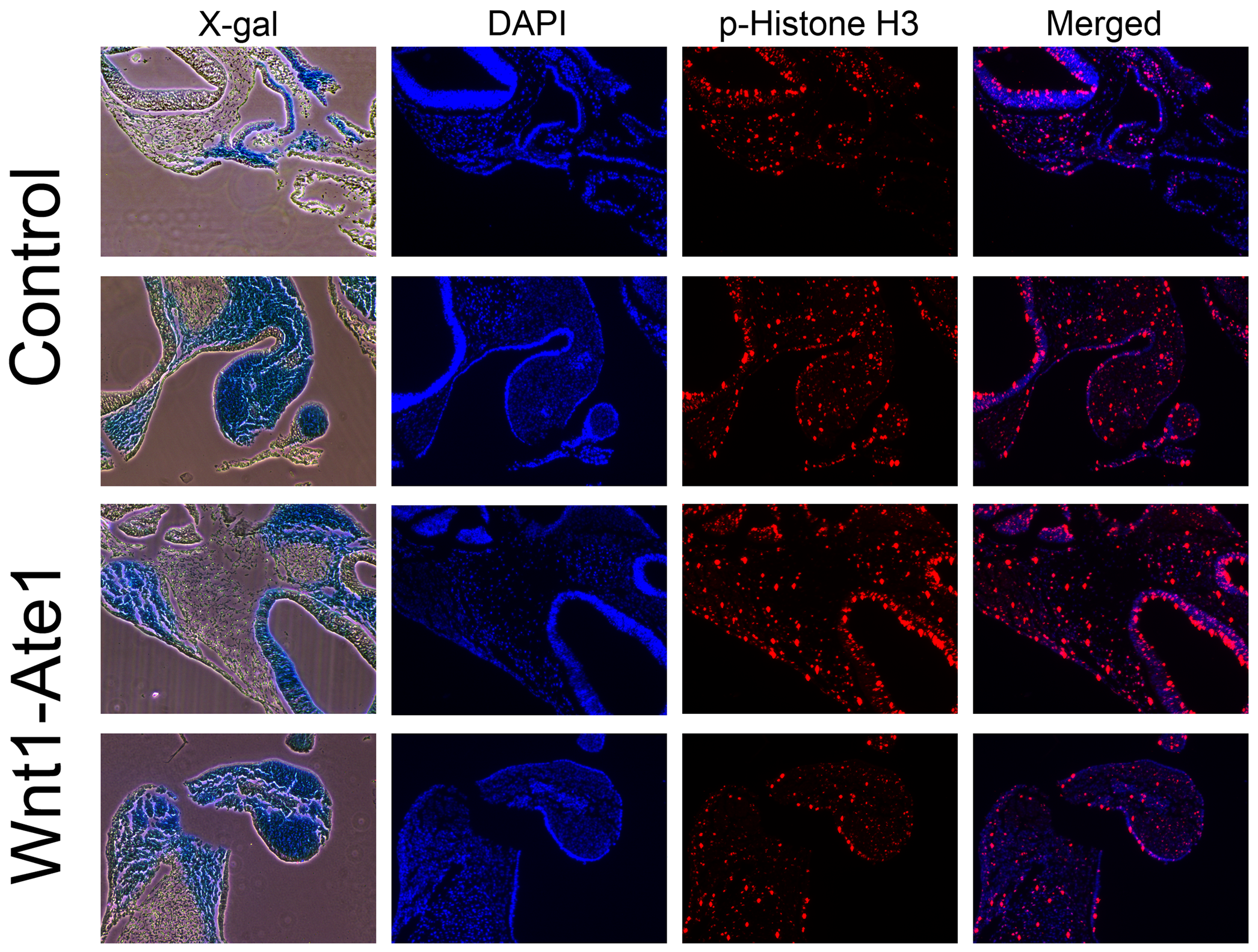

Supplement: Figure S9 — Ate1 knockout does not affect neural crest cell proliferation rates. X-gal stained E9.5 wild-type (top) and Wnt1-Ate1 (bottom) embryos were sectioned and immunostained for cell proliferation marker phospho-histone H3. No differences in staining were observed between wild-type and Wnt1-Ate1 in X-gal stained tissues. (4.03 MB TIF) [file pgen.1000878.s009.tif]

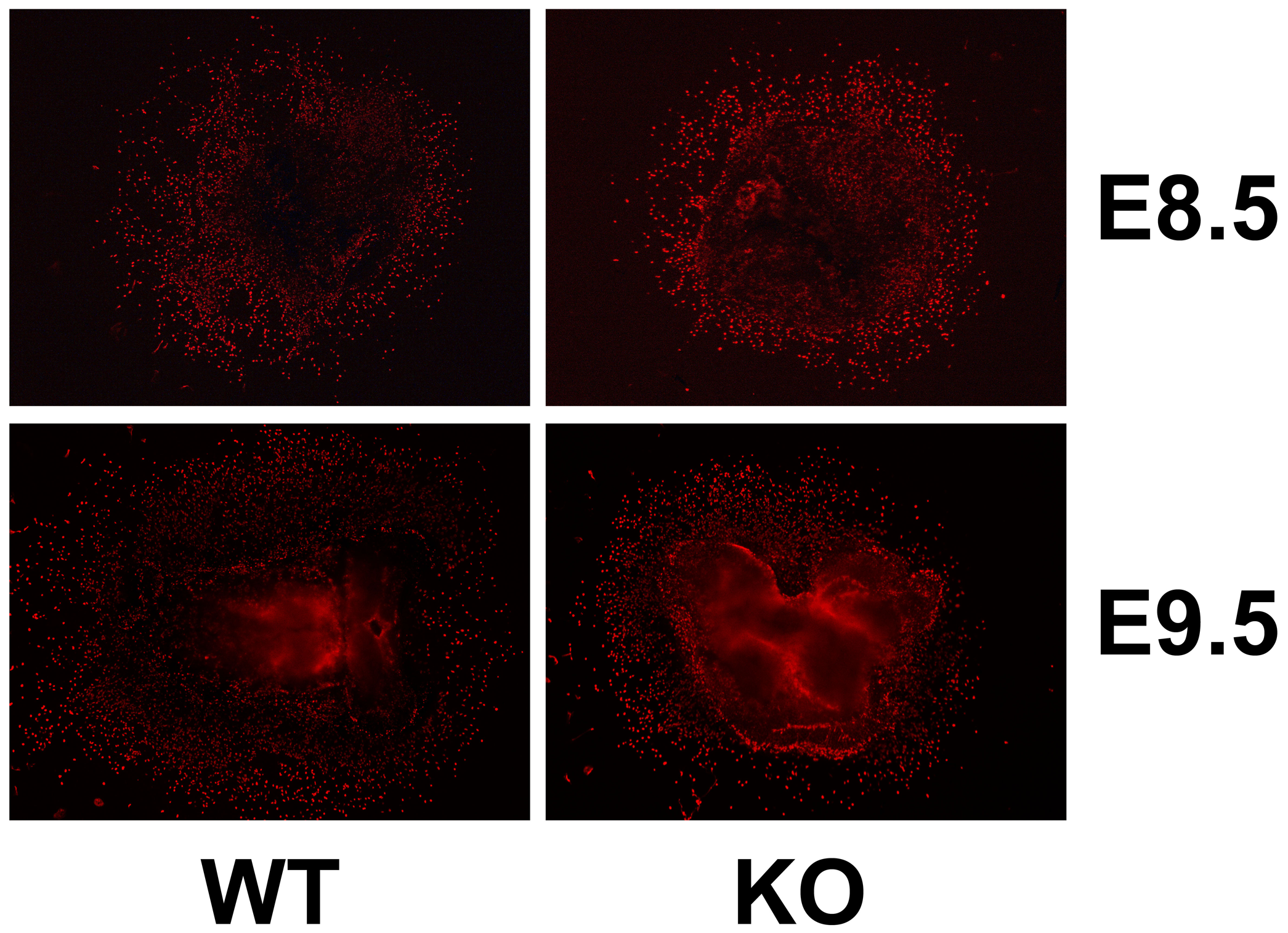

Supplement: Figure S10 — Ate1 knockout does not affect neural crest cell proliferation rates. Neural crest explants from E8.5 and E9.5 Ate1 knockout embryos labeled with BrdU after 2 days in culture. Levels of BrdU staining are similar in wild-type and knockout explants, suggesting no differences in proliferation rates of the neural crest cells. (3.57 MB TIF) [file pgen.1000878.s010.tif]

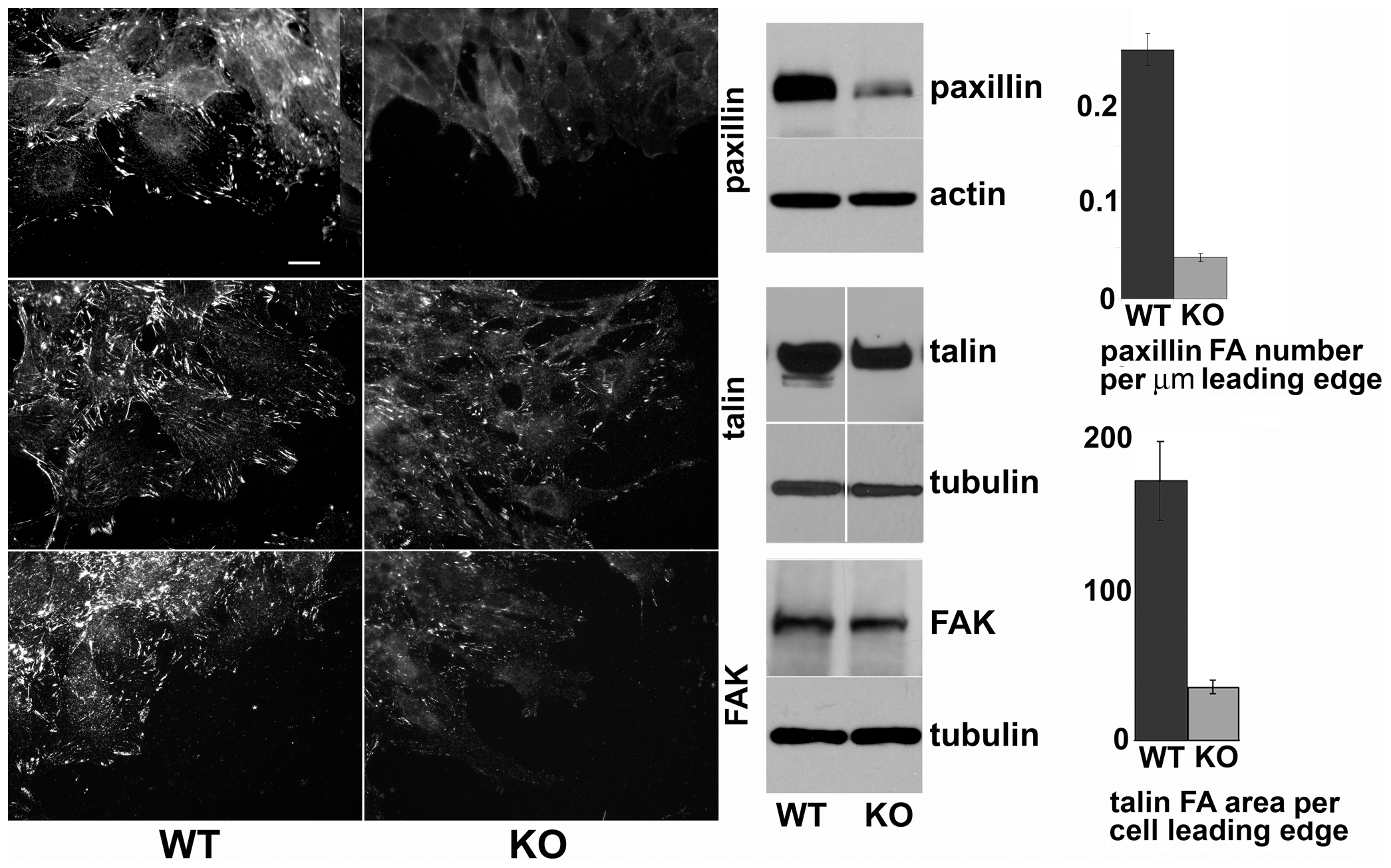

Supplement: Figure S11 — Ate1 knockout results in reduced focal adhesions. Left panels, fluorescence staining of the edge of the cell monolayer moving into the wound with anti-paxillin (top), anti-talin (middle), and anti-focal adhesion kinase (FAK, bottom) to visualize focal adhesions. Knockout cells show a dramatic reduction in focal adhesion area and number. Bar, 20 µm. Middle panels, Western Blotting comparison of the focal adhesion protein levels in wild-type and Ate1 knockout cells. Loading was adjusted by weight of the packed cell pellets and verified by loading control–actin for paxillin, and tubulin for talin and FAK, as shown in the image. Right panels, quantification of the number of paxillin focal adhesions per µm of the wound edge and of the area of talin focal adhesions per cell leading edge shows that the number of prominent focal adhesions in wild-type exceeds that in the knockout by over 5-fold. Error bars for paxillin represent SEM for the measurements in 21 and 18 different images in WT and KO, respectively. Error bars for talin represent SEM for the measurements of 15 WT and 18 KO cells. (2.18 MB TIF) [file pgen.1000878.s011.tif]

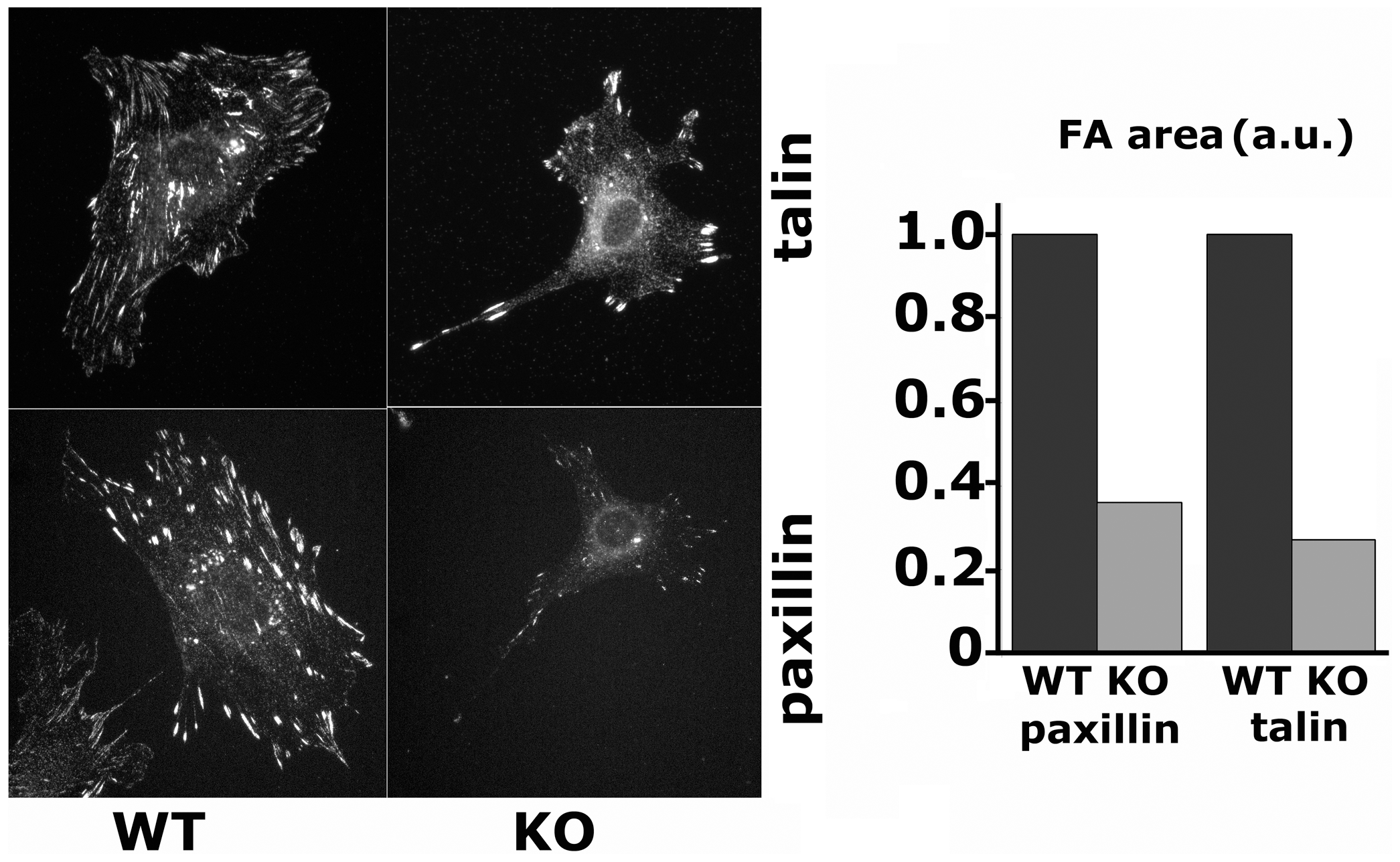

Supplement: Figure S12 — Ate1 knockout results in reduced focal adhesions. Left panels, fluorescence staining of single cells with anti-talin (top) and anti-paxillin (bottom) to visualize focal adhesions. Knockout cells show a dramatic reduction in focal adhesion area and number. Right panels, quantification of the number of focal adhesion area per cell shows that the number of prominent focal adhesions in wild-type exceeds that in the knockout by several fold. Quantifications shown represent measurements of 15 WT and 15 KO cells (paxillin) and 21 WT and 25 KO cells (talin). (2.07 MB TIF) [file pgen.1000878.s012.tif]

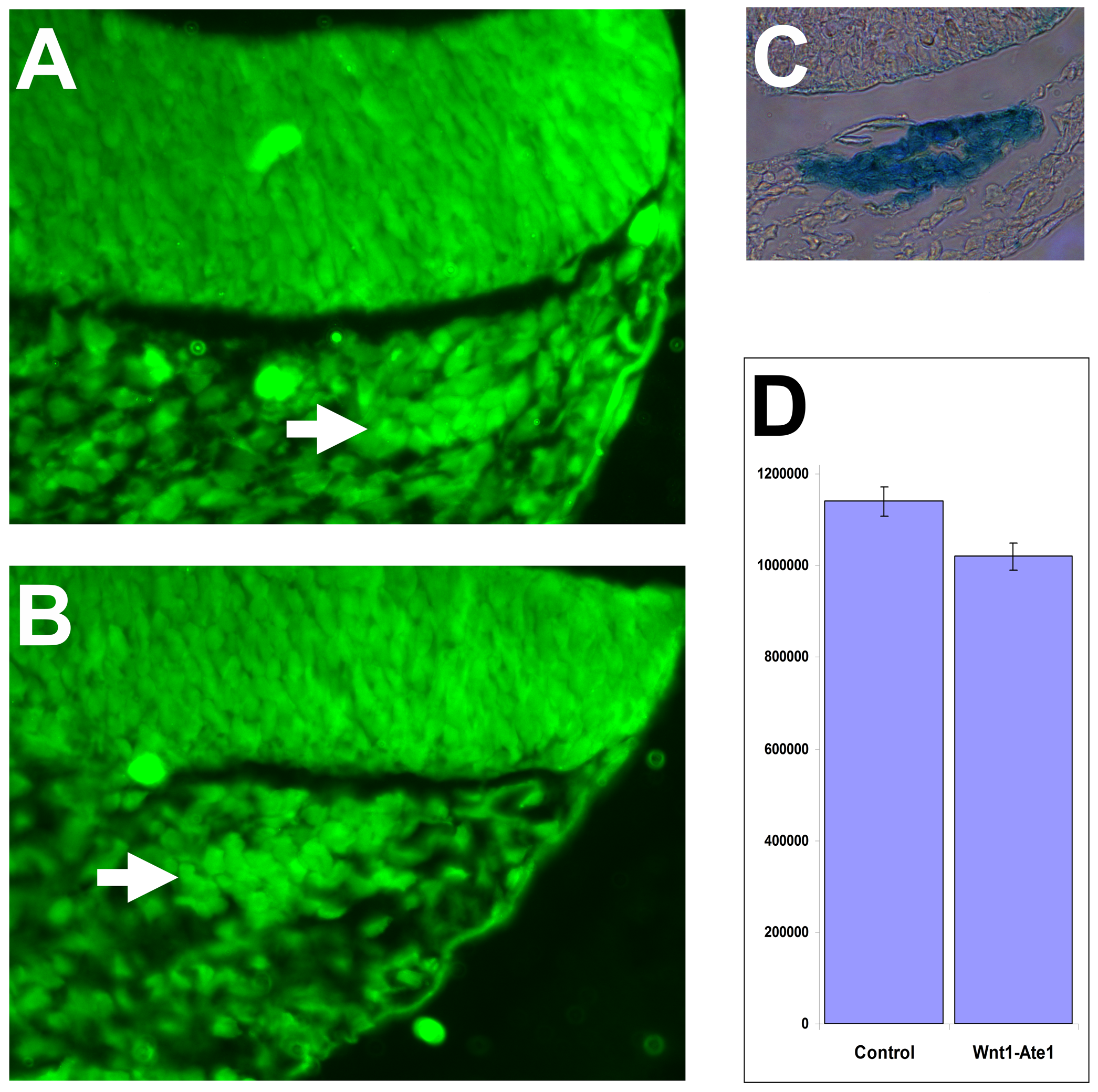

Supplement: Figure S13 — Ate1 knockout results in reduced focal adhesions. Cross sections of wild-type (A) and Wnt1-Ate1 (B) embryos at E9.5 stained with antibody to talin. C, a similar area from an X-gal stained embryo showing the location of the migratory neural crest cell population used for the quantification of talin levels shown in D. Error bars represent SEM for measurements of 6 regions taken from each of the 5 wild-type and 4 Wnt1-Ate1 sections (30 wild-type and 24 Wnt1-Ate1 measurements), p-value<0.01. (2.98 MB TIF) [file pgen.1000878.s013.tif]

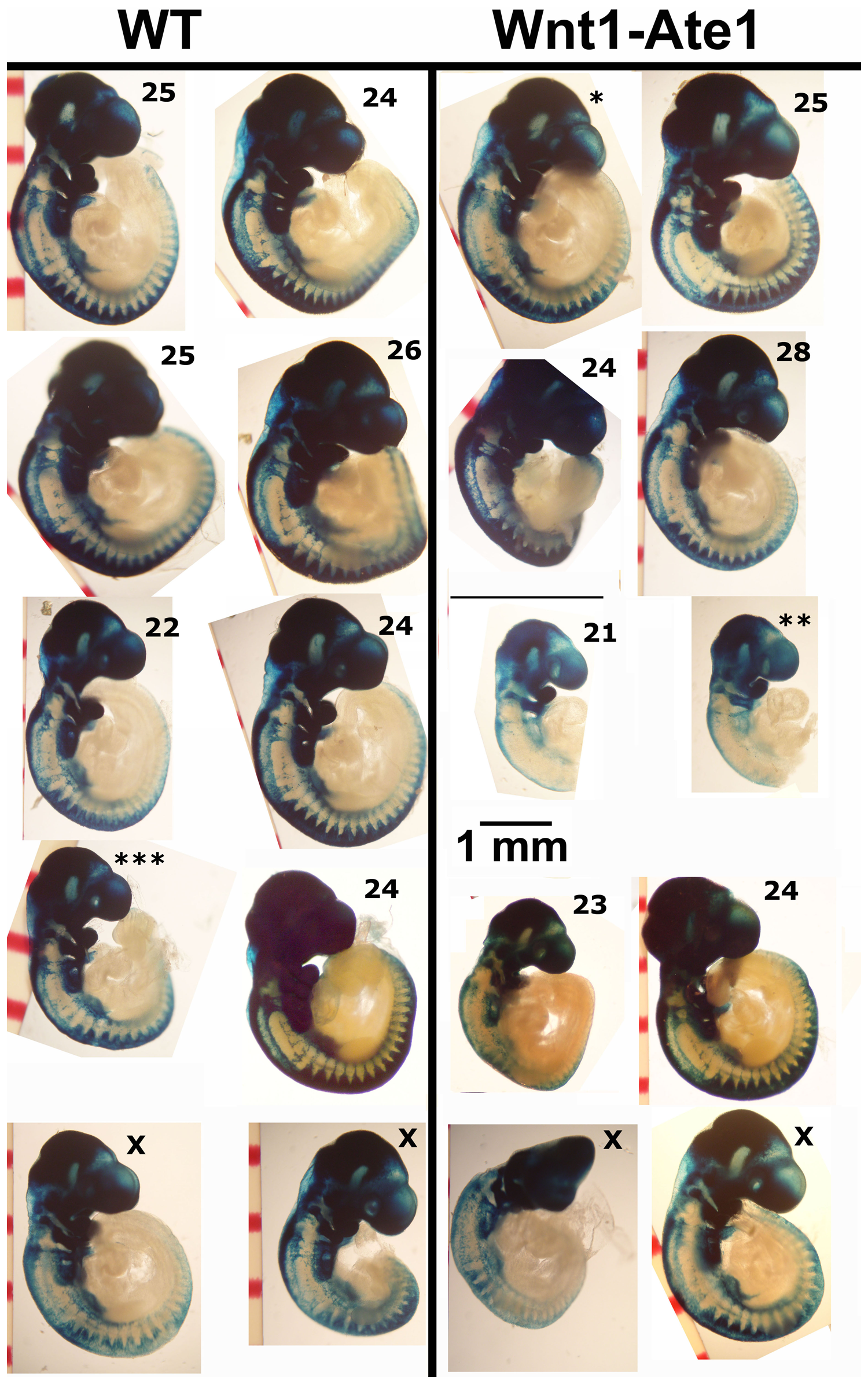

Supplement: Figure S14 — Images of X-gal-stained E9.5 wild-type (left) and Wnt1-Ate1 (right) embryos used for obtaining higher magnification views shown in Figure 4 of the main text. Embryos were staged by somite count, indicated for each embryo on the top right next to the head. For some embryos (marked with X and asterisks) somite counts were not performed. These embryos were staged by comparison with their littermates as follows: *–littermates (shown on right) had 25 and 28 somites; **–littermate (shown on left) had 21 somites; ***–littermates had 22–26 somites; X–four embryos from the same litter lined up on the bottom for comparison. (4.29 MB TIF) [file pgen.1000878.s014.tif]

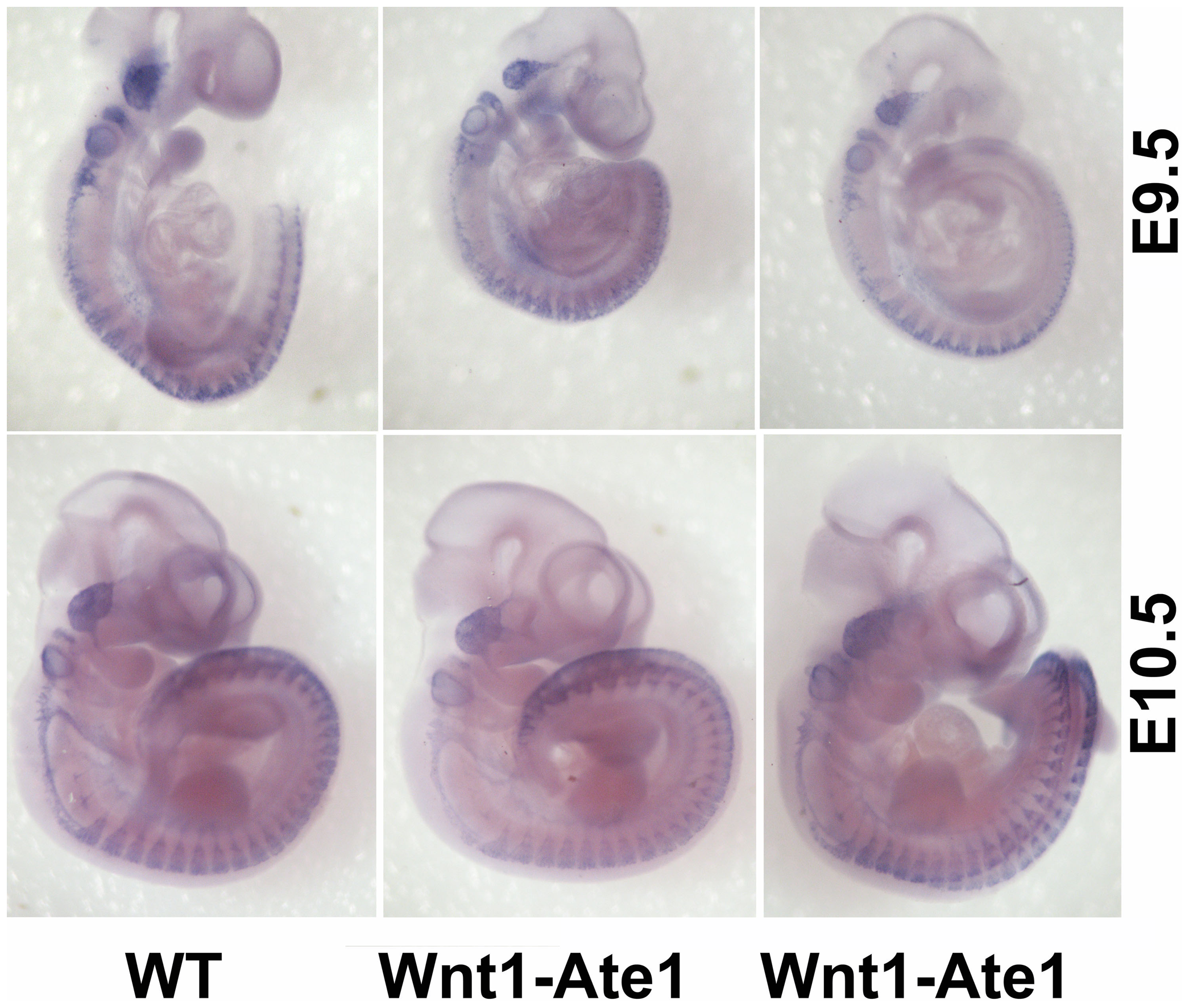

Supplement: Figure S15 — Images of Sox10-stained E9.5 and E10.5 wild-type and Wnt1-Ate1 embryos used for obtaining higher magnification views shown in Figure 4 of the main text. (4.99 MB TIF) [file pgen.1000878.s015.tif]
